# UK Medical Cannabis Registry: A Clinical Outcomes Analysis for Migraine

**DOI:** 10.1002/brb3.71323

**Published:** 2026-04-06

**Authors:** Lennon Hooper, Simon Erridge, Evonne Clarke, Katy McLachlan, Ross Coomber, Augustin Iqbal, James J. Rucker, Mark W. Weatherall, Mikael H. Sodergren

**Affiliations:** ^1^ Imperial College Medical Cannabis Research Group, Department of Surgery and Cancer Imperial College London London UK; ^2^ Curaleaf Clinic London UK; ^3^ St. George's Hospital NHS Trust London UK; ^4^ Department of Psychological Medicine Kings College London London UK; ^5^ South London & Maudsley NHS Foundation Trust London UK; ^6^ Buckinghamshire Healthcare NHS Trust Amersham UK

**Keywords:** cannabis, headache, migraine, tetrahydrocannabinol

## Abstract

**Introduction:**

Migraine is a primary headache disorder, which leads to diminished health‐related quality of life (HRQoL). There is limited evidence assessing efficacy of cannabis‐based medicinal products (CBMPs) for migraine. This study investigates the efficacy and safety of CBMPs to treat migraine headache using validated patient‐reported outcome measures.

**Methods:**

This case series utilizes data from the UK Medical Cannabis Registry. Primary outcomes included changes in Headache Impact Test 6 (HIT‐6), Migraine Disability Assessment Test (MIDAS), Generalized Anxiety Disorder 7 (GAD‐7), Single Item Sleep Quality Scale (SQS), and EuroQol 5‐Dimensions 5‐Levels (EQ‐5D‐5L) from baseline up to 24 months. Adverse events (AEs) and their severity were recorded. A *p*‐value < 0.050 was statistically significant.

**Results:**

Two hundred and three adult patients met inclusion criteria. Improvements at all intervals up to 24 months, relative to baseline, were observed in the HIT‐6, GAD‐7, SQS, and EQ‐5D‐5L (*p* < 0.010) and up to 12 months in the MIDAS (*p* < 0.050). Female sex (odds ratio [OR] 0.48, 95% confidence interval [CI] 0.23–0.98, *p* = 0.046) and a bottom quartile THC dose (OR 0.25, CI 0.06–0.93, *p* = 0.047) were negative predictors of MIDAS improvement. One in seven (*n* = 31; 15.27%) patients reported 249 AEs (*n* = 110, 44.18% mild; *n* = 79, 31.73% moderate; *n* = 57, 26.91% severe; *n* = 3, 1.20% life‐threatening).

**Discussion:**

CBMPs were associated with improvements in HRQoL measures up to 2 years, being relatively well tolerated. Higher THC doses were associated with a greater likelihood of improvement on migraine‐specific measures, although wide confidence intervals warrant caution in interpreting results.

**Conclusion:**

Findings indicate utility of CBMPs for migraine, but randomized controlled trials are required to establish causation.

## Graphical abstract

This case series followed patients receiving medicinal cannabis for migraine headache for up to 2 years. At 24 months, there were significant improvements in measures assessing headache impact, anxiety, sleep quality, and general quality of life. Note that 15.27% of patients reported an adverse event, most of which were mild or moderate in severity.

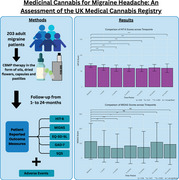

## 1 Introduction

### 1.1 Background

As of 2016, migraine was the second largest contributor to global disability and the leading cause of disability in 15–49‐year‐olds (Vos et al. 2017), causing 45.1 million years lived with disability annually (Vos et al. 2017). The Global Burden of Disease Study 2019 estimates the global prevalence to be 14.1% (Safiri et al. 2022). Characterized by moderate or severe intensity, migraine headache attacks last between 4 and 72 h, with 50% of individuals experiencing attacks on 2 or more days per month (National Institute for Health and Care Excellence 2024). Consequently, migraine imposes substantial economic and social burden, through lost productivity and reduced health‐related quality of life (HRQoL) (Linde et al. 2012; Taşkapilioğlu and Karli 2013).

### 1.2 Endocannabinoid System

The endocannabinoid system (ECS) plays an important regulatory role in physiological functions, including sleep, emotions, appetite, memory and pain (Burggren et al. 2019; Mechoulam and Parker 2013). There are two key cannabinoid receptors, the cannabinoid receptor 1 (CB_1_) and cannabinoid receptor 2 (CB_2_) (Gérard et al. 1990; Munro et al. 1993).

CB_1_ is widely distributed throughout the CNS (Svíženská et al. 2008). CB_2_ is mainly expressed in microglial cells, especially in neurodegenerative diseases (Aymerich et al. 2018), but can also be found physiologically in the brainstem and hippocampus (Stempel et al. 2016; Van Sickle et al. 2005). CB_1_ is the primary mediator of the psychoactive properties of cannabinoid ligands (Mackie 2006), and its stimulation inhibits neurotransmitter release. Two key endogenous lipids have been shown to activate CB_1_ and CB_2_: anandamide and 2‐arachidonoylglycerol (Devane et al. 1992; Sugiura et al. 1995).

### 1.3 Phytocannabinoids and Migraine

The flower of the *Cannabis sativa* L. plant contains over 560 pharmaceutical constituents, containing an array of phytocannabinoids that interact with the ECS (El‐Sohly et al. 2017). The two most abundant cannabinoids are delta‐9‐tetrahydrocannabinol (Δ9‐THC) and cannabidiol (CBD). Δ9‐THC is a partial agonist of CB_1_ and CB_2_, with a stronger affinity for CB_1_ (Boggs et al. 2016). This inhibits neurotransmitter release at both inhibitory and excitatory neurons, which may reduce the activation of afferent nociceptive fibers that trigger migraine pain and their subsequent sensitization. Migraine patients exhibit increased levels of pro‐inflammatory cytokines interictally (Fu et al. 2023; Thuraiaiyah et al. 2022), which may be attenuated by Δ9‐THC treatment. Research also demonstrates that Δ9‐THC can reduce the levels of pro‐inflammatory cytokines through stimulation of CB_2_ (Tanasescu and Constantinescu 2010; Zorrilla et al. 2024).

Conversely, CBD exhibits very low affinity for CB_1_ and CB_2_. CBD reduces anandamide catabolism and stimulates ECS activation by binding competitively to fatty acid binding proteins‐intracellular transporters of anandamide (Elmes et al. 2015). Evidence also supports its role as a negative allosteric modulator of CB_1_ (Laprairie et al. 2015). At low concentrations, CBD is an agonist of transient receptor potential vanilloid (TRPV) channels and serotonin receptors (Britch et al. 2021). As early studies observed that serotonin levels are depressed during migraine attacks (Anthony et al. 1967) and that low serotonin could trigger these events (Curzon et al. 1969; Sicuteri et al. 1976), CBD may act through this pathway. Activation of TRPV1 has been linked to release of CGRP, a neuropeptide that mediates signaling linked to migraine pain (Della Pietra et al. 2024; Meents et al. 2010). At high doses, CBD desensitizes these receptors (Anand et al. 2020; Iannotti et al. 2014). CBD may also desensitize the 5‐HT_1a_ receptors (Alexander et al. 2025), counteracting 5‐HT_1a_ hypersensitivity observed in migraine patients (Cassidy et al. 2003).

### 1.4 Cannabis‐Based Medicinal Products

A 2022 systematic review of nine studies found promising data on cannabis‐based medicinal products (CBMPs) in treating migraine, noting they appear safe and well‐tolerated (Sherpa et al. 2022). One recent randomized controlled trial (RCT), currently in pre‐print, compared vaporized cannabis to placebo for the treatment of acute migraine (Schuster et al. 2024). It showed superiority over placebo in achieving pain relief at 2 h (67.2% vs. 46.6%) as well as sustained pain freedom at 24 and 48 h, with no serious adverse events (AEs) reported (Schuster et al. 2024).

A 2020 study by Cuttler et al. (2020) assessed the acute impact of cannabis products on migraine and headache, finding that cannabis reduced migraine severity in 88.1% of 7441 use sessions among 653 patients. The study was limited, however, by biased sampling, no control of cannabis dosing, and neglect of HRQoL outcome assessment.

A 2020 Israeli, cross‐sectional, retrospective chart review by Aviram et al. (2020) found 61% of patients treated with CBMPs for migraine observed a ≥ 50% reduction in migraine frequency and that those responsive to treatment received higher phytocannabinoid doses. It was limited, however, by study design, introducing response bias and not allowing analysis of changes over time.

One previous study has used the UK Medical Cannabis Registry (UKMCR) to study the impact of CBMPs on primary headache disorders (Nicholas et al. 2023), finding improvements in multiple headache‐specific and general patient reported outcome measures (PROMs). However, this study was not migraine‐specific and only followed patients for 6 months, preventing observation of efficacy changes or tolerance development (Piscura et al. 2023).

Overall, current research is limited by short follow‐up periods, biased data collection, lack of focus on HRQoL outcomes, and wide heterogeneity of CBMP strain, dose, and administration. This leaves a clear evidence gap for a study that can analyze changes in efficacy over longer time periods using validated patient outcome measures while recording detailed prescription information.

The aim of this study is to analyze changes in HRQoL outcomes in patients treated with CBMPs for migraine in the UK, using data from the UK Medical Cannabis Registry (UKMCR). Secondary aims include assessment of the incidence of AEs and the factors associated with improvements in HRQoL.

## 2 Methods

### 2.1 Study Design

This study assesses a case series of patients with a primary diagnosis of migraine enlisted in the UKMCR. Patients provided informed and written consent prior to consecutive enrollment. Data on comorbidities, drug and alcohol history, medication use, and psychiatric history were captured from patient electronic health records. Data on PROMs, AEs, and medication changes were collected through electronic questionnaires at baseline, 1‐, 3‐, 6‐, 12‐, 18‐, and 24‐month intervals.

### 2.2 Participants and Settings

The UKMCR is a prospective registry designed to collect data from patients who are being treated with CBMPs for a variety of chronic health conditions. It was established by Curaleaf Clinic in 2019. Data are captured from patients in the United Kingdom and Crown Dependencies. Prescriptions were initiated in line with national guidance (Medicines and Healthcare Products Regulatory Agency 2020).

Patients ≥ 18 years old with a primary indication of migraine were included. A diagnosis of migraine was confirmed through primary care records alongside evidence that patients had trialled but failed to gain sufficient benefit from licensed therapies, in line with UK guidance (Case 2020). Patients were excluded if baseline PROMs were incomplete or if they were enrolled ≤ 2 years prior to data extraction on January 6, 2025.

The UKMCR has received ethical approval from the Central Bristol Research Ethics Committee (22/SW/0145).

### 2.3 Data Collection

Patient details were collected on age, sex, occupation, height, weight and body mass index (BMI). Data on an array of comorbidities were collected including asthma, cerebrovascular accidents/transient ischemic attack, hypertension, and epilepsy. Comorbidity data as well as age status were used to calculate the Charlson comorbidity index (CCI) for each patient. The scoring system of the CCI is available in Table S1.

Data on alcohol consumption, tobacco usage, and previous cannabis use were collected. Lifetime cannabis use was calculated using the gram‐years metric. Gram‐years is calculated by multiplying mean cannabis use per day by total number of years of use. Frequency of cannabis use and method(s) of cannabis consumption were gathered for current cannabis users at baseline, before CBMP prescription.

CBMP prescription data were recorded to include THC and CBD concentration, method of administration, and dosing regimen. This was utilized to calculate the dose of CBD and THC in mg/day.

Baselines PROMs were obtained using several measures: the EuroQoL 5‐Dimensions 5‐Level Version (EQ‐5D‐5L) (Herdman et al. 2011), Generalized Anxiety Disorder‐7 (GAD‐7) (Spitzer et al. 2006), and Single Item Sleep Quality Scale (SQS) (Snyder et al. 2018). Two headache‐specific measures were also used: the Headache Impact Test‐6 (HIT‐6) (Yang et al. 2011) and the Migraine Disability Assessment Test (MIDAS) (Stewart et al. 2003). Assessment details are outlined in Table 1.

**TABLE 1:** A table summarizing the different patient reported outcome measures (PROMs) used in the present study, including their scoring system, objective of assessment, range of scores, and values for minimally important clinical difference.

Patient‐reported outcome measure	Description	Score	Minimal clinically important difference
**EuroQol 5‐Dimensions 5‐Level Version (EQ‐5D‐5L)**	A quality‐of‐life assessment that assesses five domains using a single item, as well as one visual analogue scale. Domains assessed are: 1. Mobility 2. Self‐care 3. Usual activities 4. Pain/discomfort 5. Anxiety/depression Each item has five levels—no problems/issue, slight problems, moderate problems, severe problems and unable to/extreme problems. This is used to create 3125 value sets, before generating country‐specific index values. Weighting of each item is determined by value sets for different countries. A higher score reflects better quality of life.	−0.59–1	N/A
**Generalized Anxiety Disorder‐7 (GAD‐7)** (Spitzer et al. 2006)	A seven‐item questionnaire that screens for and assesses the likely severity of generalized anxiety disorder (GAD). Questions are based on diagnostic criteria for GAD from the Diagnostic and Statistical Manual of Mental Disorders, Fourth Edition. Each item scores from 0 to 4 (not at all, several days, more than half the days, nearly every day). Scores are totalled as follows: 0–4: minimal anxiety, 5–9: mild anxiety, 10–14: moderate anxiety, 15–21: severe anxiety	0–21	4‐point change (Toussaint et al. 2020)
**Patient Global Impression of Change (PGIC)**	A single item questionnaire assessing the overall change to a patient's condition since commencing treatment. The patient rates their status as: 1—no change (or condition has gotten worse), 2—almost the same, 3—a little better, 4—somewhat better, 5—moderately better, 6—better and a definite improvement, 7—a great deal better and a considerable improvement	1–7	N/A
**Single Item Sleep Quality Scale (SQS)**	A single item questionnaire that uses a scale to rate the overall quality of sleep during the past 7 days only. Patients can rate the quality of their sleep from 0 to 10, with categories outlined as followed. 0: terrible, 1–3: poor, 4–6: fair, 7–9: good, 10: excellent	0–10	2.6 (Snyder et al. 2018)
**Headache Impact Test (HIT‐6)**	A six‐item questionnaire assessing six domains affected by headaches: pain, social functioning, role functioning, cognitive function, vitality and psychological stress. Each item has five levels: never, rarely, sometimes, very often, always. Items are scored from 6 to 13, with a higher score being more severe. Total scores indicate as follows: < 49: little or no impact; 50–55: some impact; 56–59: substantial impact; 60–78: severe impact.	36–78	5‐point decrease (American Headache Society 2019)
**Migraine Disability Assessment Test (MIDAS)**	A five‐item questionnaire aiming to assess the disability consequential to migraine headache. Items assess impact to work or school, productivity, household activities, household productivity, social activities as well as frequency and pain severity. Patients score based on how many days in the last 3 months they were affected. To note, supplemental questions on headache frequency and pain severity do not score.	0–21+	If baseline score ≤ 20, absolute reduction by 5 points. If baseline score > 20, 30% reduction or more (American Headache Society 2019)

Data on AEs were collected remotely, either contemporaneously or when completing PROMs. If still incomplete, these could be recorded during consultations. AEs were graded by severity using guidance aligning with the Common Terminology Criteria for Adverse Events (CTCAE) version 4.0 (Table S2) (National Cancer Institute 2009).

### 2.4 Outcome Measures

Primary outcomes were changes in the aforementioned PROMs at 1, 3, 6, 12, 18, and 24‐months compared to baseline measurements. Patient Global Impression of Change (PGIC) questionnaires were also filled out from 1 month onward (Hurst and Bolton 2004). Secondary outcomes were the changes in PROMs between each timepoint and all other timepoints, as well as the prevalence and severity of AEs.

### 2.5 Missing Data

To account for missing PROM data in the present study, multiple imputation was used to simulate missing values. First, missing data were replaced with plausible values derived from observed data for a particular variable. Only PROMs were included in this MI model to ensure missing values were derived from patients with similar trajectories, as well as to minimize bias during logistic regression. This was conducted five times to ensure sufficient efficiency and accuracy. Each dataset is then analyzed independently before results are pooled to create final values (Haukoos and Newgard 2007; Li et al. 2015).

### 2.6 Statistical Analysis

Patient demographic data, comorbidities, alcohol and cannabis usage, and AE frequency and severity were summarized using descriptive statistics. PROMs were compared between baseline, 1‐, 3‐, 6‐, 12‐, 18‐, and 24‐month values. Significance was assessed using repeated measures analysis of variance (ANOVA) tests. For statistically significant variables, post hoc paired *t*‐tests with a Bonferroni correction were performed to reduce the chance of type I error. Univariable and multivariable logistic regression were used to assess a minimal clinically important difference in the PROMs at 24 months compared to baseline, while controlling for other variables including age, sex, BMI, previous cannabis use, CBD and THC doses, and baseline SQS and GAD scores. A *p*‐value of < 0.050 was considered statistically significant. Statistical analysis and graphical representation were conducted in R studio (Version 2024.12.1 + 563) using the R programming language (4.4.1, R Foundation for Statistical Computing, Vienna, Austria).

## 3 Results

On January 6, 2025, 34,563 patients were enrolled in the UKMCR, of which 31,508 (91.16%) had completed baseline PROMs. 22,563 (71.61%) of these patients were enrolled for at least 2 years, and 203 (0.59%) of these patients had a primary diagnosis of migraine. PROM data were missing for up to 24 (11.82%) migraine patients at 1 month, 52 (25.62%) at 3 months, 74 (36.45%) at 6 months, 91 (44.83%) at 12 months, 107 (52.71%) at 18 months, and 123 (60.59%) at 24 months (Table S3).

### 3.1 Patient Demographic Data

The mean age of patients was 39.49 ± 11.31 years. The sample contained 114 (56.16%) males and 89 (43.84%) females. Of migraine patients, 119 (58.62%) were current cannabis users (prior to prescription), 37 (18.22%) were ex‐users, and the remaining 47 (23.15%) were cannabis naïve. Further detail on demographic data is available in Table 2 and Tables S4–S7.

**TABLE 2:** A table summarizing the demographic information of the study population including age, sex, body mass index (BMI), occupation, Charlson comorbidity index score, alcohol consumption, smoking status, and cannabis status.

Variable	*n* (%)/mean ± SD/median (IQR)
Age	39.49 ± 11.31
**Sex**	

Female	89 (43.84)
Male	114 (56.16)

BMI	27.61 ± 7.73
Healthy	64 (32.65)

Obese	57 (29.08)
Overweight	56 (28.57)

Underweight	19 (9.69)
**Occupation**	

Clerical support workers	6 (2.96)
Craft and related trades workers	6 (2.96)

Elementary occupations	8 (3.94)
Managers	17 (8.37)

NR	18 (8.87)
Other occupations	35 (17.24)

Plant and machine operators, and assemblers	2 (0.99)
Professional	41 (20.20)

Service and sales workers	7 (3.45)
Skilled agricultural, forestry and fishery workers	1 (0.49)

Technicians and associate professionals	19 (9.36)
Unemployed	43 (21.18)

Charlson comorbidity index score	0.00 (0.00–0.00)
**Alcohol units per week**	0.00 (0.00–0.00)

Smoking status	
Current smoker	42 (20.69)

Ex‐smoker	81 (39.90)
Never smoked	80 (39.41)

Cannabis status	
Current user	119 (58.62)

Ex‐user	37 (18.23)
Never used	47 (23.15)

Abbreviations: IQR, interquartile range; NR, not recorded.

### 3.2 CBMP Prescription

**FIGURE 1:**
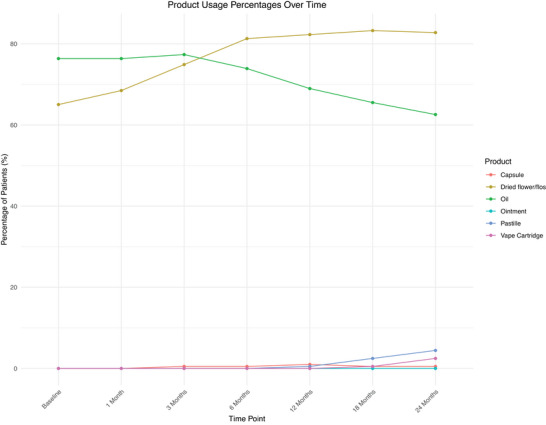
Graphical representation of product use percentages over time. A graph showing the change in percentage use of different types of cannabis‐based medicinal products over time, at baseline, 1, 3, 6, 12, 18, and 24 months.

At baseline, 71 (34.98%), 48 (23.64%), and 84 (41.38%) patients were prescribed oils, dried flower for vaporization, or both products, respectively (Figure 1).

Partway through this study, new CBMPs became available for prescription in the form of ointments, pastilles, and vape cartridges. At 24 months in the studied cohort, nine (4.43%) patients were using pastilles, and five (2.46%) patients were using vape cartridges (Figure 1). Pastilles and vape cartridges were always prescribed in combination with other CBMP products.

**FIGURE 2:**
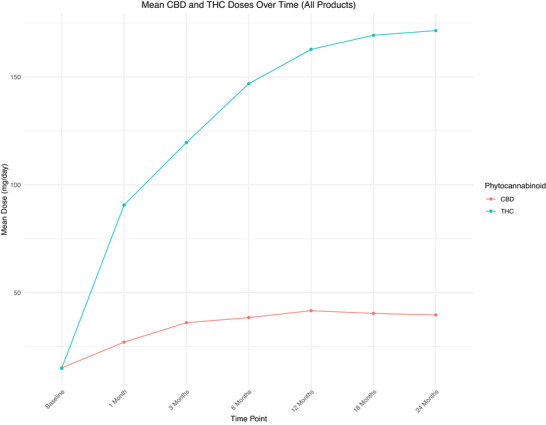
Graphical representation of mean CBD and THC dose across all timepoints. A graph showing the mean dose (mg/day) of cannabidiol (CBD) and delta‐9‐tetrahydrocannabinol (THC) at baseline, 1, 3, 6, 12, 18, and 24 months post initial cannabis‐based medicinal product (CBMP) prescription.

**FIGURE 3:**
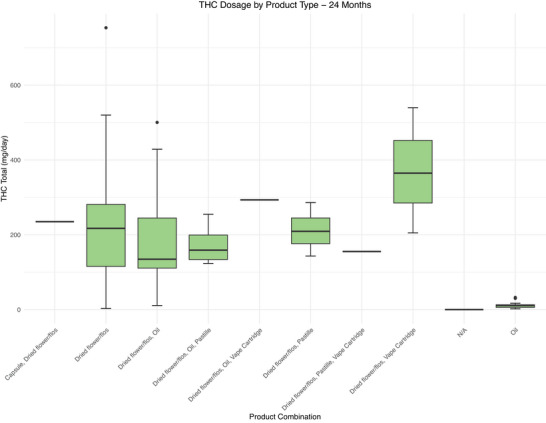
Boxplots of the distribution of THC dose for different product combinations at 24 months post initial prescription. A: Box plots showing the median, interquartile ranges, 1.5x standard deviation, and the outliers of daily THC dose in mg/day across the different product combinations that patients received. Patients in the N/A category did not have any active prescriptions at that timepoint. THC, tetrahydrocannabinol.

**FIGURE 4:**
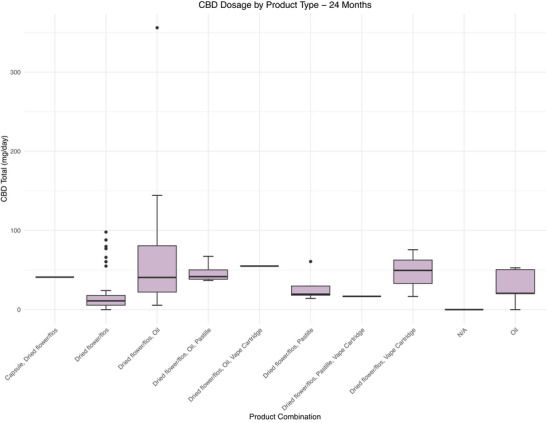
Boxplots of the distribution of CBD dose for different product combinations at 24 months post initial prescription. B: Box plots showing the median, interquartile ranges, 1.5x standard deviation, and the outliers of daily CBD dose in mg/day across the different product combinations that patients received. Patients in the N/A category did not have any active prescriptions at that timepoint. CBD, cannabidiol.

Across all products, median CBD and THC dose was 20.00 (2.00–20.10) mg/day and 19.00 (2.60–21.00) mg/day at baseline and 25.50 (11.00–58.68) mg/day and 134.00 (104.63‐249.50) mg/day at 24 months, respectively (Figure 2). Details on CBD and THC dose stratified by product are available in Figures 3, 4 and Tables S8 and S9.

### 3.3 Adverse Events

During this study, 31 (15.27%) patients reported 249 separate AEs. The most common AE was headache (*n* = 22, 10.84%), followed by dry mouth (*n* = 19, 9.36%), impaired concentration (*n* = 16, 7.88%), fatigue (*n* = 16, 7.88%), nausea (*n* = 14, 6.90%), and lethargy (*n* = 12, 6.40%). Of all AEs, 110 (44.18%) were classed as mild, 79 (31.73%) as moderate, 57 (26.91%) as severe, and three (1.20%) as life‐threatening/disabling. The three life‐threatening/disabling AEs reported were somnolence, delirium, and confusion. Full data on the frequency of AEs and their severities are available in Table 3.

**TABLE 3:** A table representing the incidence of different adverse events by count, percentage of total adverse events (AEs), and their severity (in alignment with CTCAE 4.0 criteria). Percentage was calculated in relation to total number of AEs as opposed to total patients, as each patient could report multiple AEs patients self‐reported symptoms, which were then coded by a clinician.

Adverse event	Count	Percentage (%)	Mild	Moderate	Severe	Life threatening/disabling
Headache	22	10.84	3	6	13	0
Dry mouth	19	9.36	15	4	0	0
Concentration impairment	16	7.88	12	3	1	0
Fatigue	16	7.88	6	7	3	0
Nausea	14	6.90	7	6	1	0
Lethargy	13	6.40	6	7	0	0
Insomnia	12	5.91	5	4	3	0
Dizziness	11	5.42	5	1	5	0
Somnolence	11	5.42	0	9	1	1
Constipation	9	4.43	7	2	0	0
Dyspepsia	9	4.43	3	4	2	0
Amnesia	6	2.96	3	1	2	0
Blurred vision	6	2.96	0	0	6	0
Cognitive disturbance	6	2.96	3	1	2	0
Delirium	6	2.96	5	0	0	1
Generalized muscle weakness	6	2.96	2	3	1	0
Vertigo	6	2.96	2	0	4	0
Anorexia	5	2.46	3	2	0	0
Ataxia	5	2.46	4	0	1	0
Confusion	5	2.46	4	0	0	1
Pharyngitis	5	2.46	0	4	1	0
Other (single occurrences)	5	2.46	2	2	1	0
Abdominal pain	4	1.97	1	1	2	0
Vomiting	4	1.97	3	1	0	0
Weight loss	4	1.97	3	1	0	0
Dysgeusia	3	1.48	1	1	1	0
Fever	3	1.48	1	0	2	0
Lung infection	3	1.48	0	3	0	0
Urinary tract infection	3	1.48	0	2	1	0
Cough	2	0.99	2	0	0	0
Migraine	2	0.99	0	0	2	0
Rash NOS	2	0.99	0	2	0	0
Spasticity	2	0.99	0	2	0	0
Tremor	2	0.99	1	0	1	0
Wheezing	2	0.99	1	0	1	0

Abbreviation: NOS, not otherwise specified.

### 3.4 Patient Reported Outcome Measures

Repeated measures ANOVA revealed changes across time in all PROMs (*p* < 0.001) (Table 5).

**FIGURE 5:**
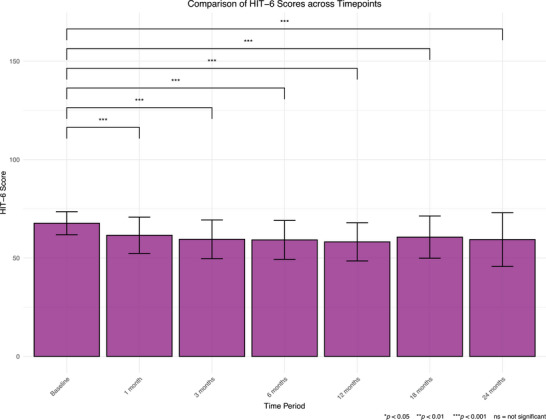
Graph showing mean scores at baseline, 1, 3, 6, 12, 18, and 24‐months in the HIT‐6. HIT‐6, Headache Impact Test 6. Whiskers represent standard deviation. *p < 0.050, **p < 0.010, ***p < 0.001, ns = not significant.

Pairwise comparisons revealed that mean HIT‐6 scores decreased at all timepoints (*p* < 0.001) compared to baseline (Figure 5). There were also decreases from 1 month to 3, 6, and 12 months (*p* < 0.010). At 18 months, scores had increased compared to 3, 6, and 12 months (*p* < 0.001). An MCID was observed in 99 patients (48.77%) at 24 months (Table 4).

**TABLE 4:** A table showing the number and percentage of patients that achieved a significant change or positive improvement in score in the different patient reported outcome measures.

Outcome Measure	1‐month significant change, *n* (%)	3‐month significant change, *n* (%)	6‐month significant change, *n* (%)	12‐month significant change, *n* (%)	18‐month significant change, *n* (%)	24‐month significant change, *n* (%)
**HIT‐6**						

Yes	105 (51.72)	126 (62.07)	132 (65.02)	138 (67.98)	122 (60.10)	110 (54.19)
No	98 (48.28)	77 (37.93)	71 (34.98)	65 (32.02)	81 (39.90)	93 (45.81)

MIDAS						
Yes	111 (54.68)	134 (66.01)	131 (64.53)	129 (63.55)	125 (61.58)	108 (53.20)
No	92 (45.32)	69 (33.99)	72 (35.47)	74 (36.45)	78 (38.42)	95 (46.80)
**GAD‐7**						
Yes	73 (35.96)	70 (34.48)	76 (37.44)	76 (37.44)	79 (38.92)	79 (38.92)
No	130 (64.04)	133 (65.52)	127 (62.56)	127 (62.56)	124 (61.08)	124 (61.08)
**SQS**						
Yes	63 (31.03)	72 (35.47)	71 (34.98)	81 (39.90)	87 (42.86)	96 (47.29)
No	140 (68.97)	131 (64.53)	132 (65.02)	122 (60.10)	116 (57.14)	107 (52.71)
	**1‐month positive improvement, *n* (%)**	**3‐month positive improvement, *n* (%)**	**6‐month positive improvement, *n* (%)**	**12‐month positive improvement, *n* (%)**	**18‐month positive improvement, *n* (%)**	**24‐month positive improvement, *n* (%)**
**EQ‐5D‐5L index value**						
Yes	136 (67.00)	137 (67.49)	136 (67.00)	136 (67.00)	132 (65.02)	136 (67.00)
No	67 (33.00)	66 (32.51)	67 (33.00)	67 (33.00)	71 (34.98)	67 (33.00)

Abbreviations: EQ‐5D‐5L, EuroQol‐5 Dimensions‐5 Levels; GAD‐7, Generalized Anxiety Disorder‐7; HIT‐6, Headache Impact Test 6; MIDAS, Migraine Disability Assessment Test; SQS, Single Item Sleep Quality Scale.

**FIGURE 6:**
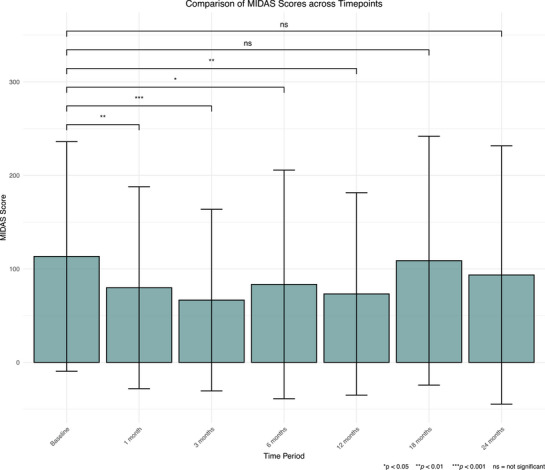
Graph showing mean scores at baseline, 1, 3, 6, 12, 18, and 24 months in the MIDAS. MIDAS, Migraine Disability Assessment Test. Whiskers represent standard deviation. *p < 0.050, **p < 0.010, ***p < 0.001, ns = not significant.

Compared to baseline, mean MIDAS scores decreased at 1, 3, 6, and 12 months (*p* < 0.050), but not at 18 or 24 months (*p* > 0.050) (Figure 6). Furthermore, mean scores at 18 months were higher than at 1, 6, and 12 months (*p* < 0.050). An MCID was seen in 128 patients (63.05%) at 24 months.

Relative to baseline, mean EQ‐5D‐5L index values increased at all timepoints (*p* < 0.001), reflecting improved perceived quality of life. A positive change was observed in 136 (67.00%) patients at 24 months. EQ‐5D‐5L dimension findings are outlined below.

Mean pain/discomfort scores improved at all intervals (*p* < 0.001). Anxiety/depression scores improved at 1, 3, 6, and 12 months (*p* < 0.001) but not at 18 and 24 months (*p* > 0.050). Usual activities scores improved at 1 month (*p* < 0.001), but not at any other timepoints. Notably, mean mobility scores worsened at 12 and 24 months versus baseline (*p* < 0.001). Self‐care scores had also decreased at 18 and 24 months (*p* < 0.010) relative to baseline.

Mean GAD‐7 scores improved from baseline at all intervals (*p* < 0.010). An MCID was observed in 79 (38.92%) patients at 24 months.

Mean SQS scores also improved at all intervals compared to baseline (*p* < 0.001). MCID was observed in 96 patients (47.29%) at 24 months.

The mean PGIC score at 1 month was 5.32 (standard deviation [SD] ± 1.52). Scores were also reported at 3 (mean = 5.75 ± 1.28), 6 (5.63 ± 1.30), 12 (5.86 ±1.22), 18 (5.97 ± 0.93), and 24 months (5.80 ±1.22). Mean score and significant change data for PROMs are available in Table 5 and Tables S10–S19.

**TABLE 5:** A table demonstrating the mean values and standard deviation for the different patient reported outcome measures and the results of the repeated measures analysis of variance (ANOVA).

PROM	Baseline mean value ± SD	1‐month mean value ± SD	3‐month mean value ± SD	6‐month mean value ± SD	12‐month mean value ± SD	18‐month mean value ± SD	24‐month mean value ± SD	Repeated measures ANOVA *p*‐value
HIT‐6	67.53 ± 5.89	62.34 ± 8.73	59.95 ± 9.63	59.37 ± 9.88	58.62 ± 10.20	63.06 ± 11.55	61.25 ± 13.22	*p* < 0.001
**MIDAS**	113.30 ± 122.79	79.86 ± 108.00	66.62 ± 97.13	83.39 ± 122.29	73.21 ± 108.27	108.79 ± 133.11	93.48 ± 138.15	*p* < 0.001

EQ‐5D‐5L	0.53 ± 0.31	0.67 ± 0.27	0.66 ± 0.26	0.68 ± 0.25	0.65 ± 0.24	0.66 ± 0.25	0.67 ± 0.29	*p* < 0.001
**GAD‐7**	7.69 ± 6.00	5.06 ± 4.67	5.36 ± 5.01	5.87 ± 5.03	5.81 ± 5.30	5.45 ± 4.60	5.42 ± 5.86	*p* < 0.001

SQS	4.68 ± 2.49	6.22 ± 2.52	6.36 ± 2.50	6.09 ± 2.46	6.39 ± 2.71	6.67 ± 2.50	6.81 ± 3.02	*p* < 0.001
**PGIC**		5.33 ± 1.52	5.75 ± 1.28	5.63 ± 1.30	5.86 ± 1.22	5.97 ± 0.93	5.80 ± 1.22	

Abbreviations: EQ‐5D‐5L, EuroQol‐5 Dimensions‐5 Levels; GAD‐7, Generalized Anxiety Disorder‐7; HIT‐6, Headache Impact Test 6; MIDAS, Migraine Disability Assessment Test; SD, standard deviation; SQS, Single Item Sleep Quality Scale.

### 3.5 Logistic Regression

Both univariable and multivariable regression models were used to assess factors associated with changes in the PROMs at 24 months.

#### 3.5.1 HIT‐6

Logistic regression did not reveal any factors that were associated with improvements in HIT‐6 scores after univariable or multivariable analysis (*p* > 0.050).

#### 3.5.2 MIDAS

Univariable logistic regression revealed that females were less likely to achieve an MCID in the MIDAS compared to males (odds ratio [OR] 0.47, 95% confidence interval [CI] 0.26–0.82, *p* = 0.008). Those vaporizing flowers only were more likely to see benefit than those using both flowers and oil (OR 2.78, 95% CI 1.17–6.83, *p* = 0.022). Those in the bottom quartile of daily THC dose were less likely to see benefit than those in the second quartile (OR 0.34, 95% CI 0.15–0.75, *p* = 0.008), third quartile (OR 0.41, 95% CI 0.18–0.91, *p* = 0.030), and upper quartile (OR 0.27, 95% CI 0.12–0.61, *p* = 0.002).

After multivariable regression, the female sex was still associated with a lower chance of an MCID relative to males (OR 0.48, 95% CI 0.23–0.98, *p* = 0.046). Compared to those in the bottom quartile of daily THC dose, only those in the top quartile had an increased chance of improvement (OR 4.03, 95% CI 1.07–17.31, *p* = 0.047).

#### 3.5.3 Adverse Events

Univariable regression did not reveal any factors that were associated with an increased or decreased risk of reporting at least one AE (*p* > 0.050).

However, multivariable regression did reveal that a BMI ≥ 35 kg/m^2^ was associated with an increased risk of reporting an AE relative to those with a BMI of 20–24.99 kg/m^2^ (OR 9.21, 95% CI 1.80–54.34, *p* = 0.010). Patients using oils alone were less likely to report an AE than patients using dried flowers only (OR 0.055, 95% CI 0.0036–0.58, *p* = 0.022) and those using dried flowers and oils in combination (OR 0.047, 95% CI 0.0038–0.370, *p* = 0.007). Patients in the bottom quartile of daily THC dose were more likely to report an AE than those in the second quartile (OR 12.50, 95% CI 2.27–100, *p* = 0.006), third quartile (OR 8.33, 95% CI 1.61–50, *p* = 0.013), and upper quartile (OR 5.88, 95% CI 1.15–33.33, *p* = 0.038).

Full details of univariable and multivariable logistic regression analysis for all PROMs are available in Tables S20–S32.

## 4 Discussion

### 4.1 Overall Findings

The findings of this study indicate that CBMPs are associated with an improvement in metrics assessing headache impact, anxiety, sleep quality, and overall HRQoL. This is indicated by improvements from baseline at all intervals for the HIT‐6, EQ‐5D‐5L, GAD‐7, and SQS, as well as up to 12 months in the MIDAS.

### 4.2 The Impact of CBMPs on Migraine

The present study's findings build upon the findings of Nicholas et al. (2023), who previously used the UKMCR to assess the use of CBMPs in headache disorders. They observed similar improvements in PROMs over the 6‐month course of their study. However, the present findings highlight a sustained improvement over 24 months. It is important to note that a downward trend was observed in those achieving an MCID in the HIT‐6 and MIDAS, despite uptitration of CBD and THC doses. This may suggest a tolerance effect (Piscura et al. 2023), echoing findings previously reported by Cuttler et al. (2020) during their assessment of CBMPs as an acute migraine treatment. Without an untreated control cohort, it is not possible to determine whether this trend reflects pharmacological tolerance or natural variation in disease course.

MIDAS scores were not significantly reduced at 18 or 24 months. This may be a result of the increased variance within the MIDAS scoring, reducing power to detect a difference (Figure 6, Table 5). Alternatively, it may reflect the differences in the questionnaires themselves. For example, the HIT‐6 has been shown to be more sensitive to headache intensity, whereas the MIDAS is more sensitive to frequency (Sauro et al. 2010). Interestingly, using cannabis before starting CBMP treatment was not associated with a reduced chance of improvement in any metric apart from SQS—which might be expected given cannabis exposure provides opportunity to develop tolerance (Piscura et al. 2023). This is supported by one paper, which found no difference in duration of cannabis use between Israeli patients who did and did not respond to CBMPs (Aviram et al. 2020). Tolerance effects could have been masked for several reasons. First, there are positive psychological effects and reduced stress from switching to a legally prescribed product. Previous users may also experience an enhanced placebo effect, stimulated by positive experiences and heightened expectancy (Gedin et al. 2022). Overall, positive effects of CBMPs for migraine appear sustained at 2 years.

Analysis of MIDAS scores revealed several predictors of outcomes. First, males were more likely to see an MCID than females, supported by evidence from two other clinical studies (Cuttler et al. 2020; Stith et al. 2020). One possibility is a differential tolerance response to cannabinoids—female rats have demonstrated heightened tolerance effects (Wakley et al. 2014). Since males were also overrepresented in this population, effect size in the MIDAS is potentially overstated relative to the general migraine population. A positive predictor of improvement in the MIDAS was a high THC content. To our knowledge, this is the first clinical study to demonstrate this in a migraine‐specific population, supported by evidence from one preclinical paper (Kandasamy et al. 2018). These findings support the tentative use of higher THC doses, given appropriate monitoring and supervision. Future studies should incorporate instruments to better assess for specific AEs that may occur with higher levels of THC.

### 4.3 Adverse Events

Thirty‐one (15.27%) patients reported 249 AEs, a majority of which were classed as mild or moderate. This incidence is slightly less than that reported by Nicholas et al. (2023) (17.5%) and lower than findings of a 2022 systematic review of CBMPs for chronic pain by Zeraatkar et al. (2022) at 26% prevalence. It is also lower than that reported in other UKMCR cohorts, including those with inflammatory arthritis (25.61%; Francis et al. 2025) and PTSD (20.37%; Pillai et al. 2022). There were 57 reports of severe AEs and three life‐threatening/disabling reports of somnolence, confusion, and delirium. However, the proportion of AEs graded as severe (26.91%) was higher than that observed in the UKMCR inflammatory arthritis cohort (13.48%; Francis et al. 2025), where comparable CTCAE 4.0 methodology was used. Of note, the CTCAE definition of “severe” encompasses events that are medically significant but not immediately life‐threatening, including those limiting self‐care activities of daily living, and therefore does not equate to a “serious” AE as defined by regulatory frameworks. In long‐term observational CBMP studies for chronic non‐cancer pain, serious AEs have been reported in approximately 3.0% of patients (Bialas et al. 2022). AEs in the present study were not assessed as to whether they were treatment‐related, precluding definitive attribution of these events to CBMPs. The relatively high proportion of severe‐graded events underscores the need for standardized AE monitoring across CBMP studies to enable meaningful cross‐study comparison and inform safe prescribing practices. Headache was the most prevalent adverse effect (*n* = 22, 10.84%), reported at a higher incidence than most other CBMP literature, specific to migraine or not (Baraldi et al. 2022; Erridge et al. 2021; Nicholas et al. 2023; Rhyne et al. 2016). It does support findings from the previous UKMCR headache disorder study, which found an 11.3% incidence of headache as an AE. These reports may have been due to priming of patients to report headaches they experience usually; worsening of headache symptoms consequential to CBMP treatment; and expectation bias affecting patients who were not educated regarding headache burden and AE reporting. However, the study design meant it was not possible to distinguish between these. Future CBMP literature should heed close attention to headache as an AE in migraine populations.

Multivariable logistic regression suggested that both using oils alone and using a high dose of THC were associated with reduced odds of experiencing an AE. Contrastingly, patients using oils usually have low THC doses. There are several possible reasons for this. First, a form of survivorship bias may occur, where patients not experiencing AEs are more likely to seek higher doses of THC compared to those experiencing AEs, who may be resultantly hesitant. Different formats, such as vaporized dried flower, may be associated with increased risk of side effects related to administration, rather than the constituent active ingredients. However, as the use of oils and a low THC dose are likely highly correlated, also known as multicollinearity, the regression model may struggle to determine which variable is correlated with the effect, producing unreliable results (Midi et al. 2010). Therefore, these findings should be treated with caution.

### 4.4 Limitations

There are several limitations of this study that should be addressed. First, the case series design of the present study limits interpretation of findings to associations and cannot be used to establish cause and effect. Additionally, the lack of a control group and blinding means that it is impossible to determine whether benefits were a result of CBMP use or other factors, including placebo effects and regression to the mean. As a private clinic and prescription service, a financial barrier likely existed to receiving treatment, leading to selection bias. However, the most common employment status in the study population was “unemployed,” suggesting that patients with a lower socioeconomic status were represented. Moreover, paying for treatment may enhance the placebo effect, as cost can be associated with perceived benefit (Lee et al. 2020).

Despite only making up 7.4% of the UK population (Office for National Statistics 2024), current cannabis users made up over half of the study population. This trend is consistent across the UKMCR (Erridge et al. 2021) and reflects that current cannabis users are more likely to access CBMP treatment. Migraineurs are more commonly female (Vetvik and MacGregor 2017), but males were overrepresented in this study, making up 56% of patients. This was likely because there are more male cannabis users overall (Mattingly et al. 2024) and that current users were more likely to access CBMPs. The sex‐specific differences, as well as the overall outcomes, should therefore be interpreted cautiously considering this selection bias.

Results pertaining to cannabinoid doses should also be interpreted carefully. Oils, flower vaporization, pastilles, and capsules have differing phytocannabinoid bioavailability (Radparvar 2024). Hence, it is unlikely that equivalent prescribed doses result in similar or identical serum plasma concentrations, complicating analysis.

By the end of the present study, PROM data were available for around 120 out of 203 patients. A dropout rate of around 60% is not uncommon within CBMP literature (Nicholas et al. 2023; Rhyne et al. 2016). High patient attrition was likely due to a variety of reasons, including lack of efficacy and adverse effects, patients switching clinics, and financial reasons. This study accounted for missing data using multiple imputation, which relies on data being missing at random (MAR) (Li et al. 2015). If data are missing not at random (MNAR)—if they are dependent on the unobserved data—results of imputation can become biased. In the present study, it is likely that data falls somewhere between MAR and MNAR, meaning some bias may have been introduced.

## 5 Conclusion

The findings of this study show that CBMPs are associated with improved HRQoL over 24 months in both headache‐specific and other PROMs. While AEs did occur during treatment, the majority of these were mild or moderate in severity. A higher THC dose also appears to be associated with increased chance of improvement. While RCTs are necessary to confirm the observed associations, this study may serve to guide THC and CBD dosing for future studies and reinforce the importance of structured AE recording.

## Author Contributions

All authors contributed to the research conceptualization. Data were curated by Lennon Hooper, Simon Erridge, Evonne Clarke, Katy McLachlan, Ross Coomber, Augustin Iqbal, James J. Rucker, and Mark W Weatherall. Formal analysis was conducted by Lennon Hooper, Simon Erridge, and Mikael H. Sodergren. Study investigation was conducted by Lennon Hooper, Simon Erridge, and Mikael H. Sodergren. Methodological development was performed by Lennon Hooper, Simon Erridge, Ross Coomber, James J. Rucker, Mark W Weatherall, and Mikael H. Sodergren. Visualization of data was synthesized by Lennon Hooper, Simon Erridge, and Mikael H. Sodergren. Writing of the original draft was performed by Lennon Hooper, Simon Erridge, and Mikael H. Sodergren. All authors contributed to the reviewing and editing of the manuscript.

## Funding

The authors have nothing to report.

## Ethics Statement

Ethical approval was provided by the Central Bristol Research Ethics Committee. Reference number: 22/SW/0145.

## Conflicts of Interest

Lennon Hooper has no conflicts of interest. Simon Erridge is Research Director at Curaleaf Clinic. Evonne Clarke is the Patient Care Director at Curaleaf Clinic. Katy McLachlan is Chief Pharmacist at the Curaleaf Clinic. Ross Coomber is the Operations Director at Curaleaf Clinic. Augustin Iqbal is a consultant in neurorehabilitation at Curaleaf Clinic. James J. Rucker is a consultant psychiatrist at Curaleaf Clinic. James J. Rucker is funded by a fellowship (CS‐2017‐17‐007) from the National Institute for Health Research (NIHR). Mark W Weatherall is a consultant neurologist at Curaleaf Clinic. Mikael H. Sodergren is the Chief Medical Officer at Curaleaf International. The authors have no other relevant affiliations or financial involvement with any organization or entity with a financial interest in or financial conflict with the subject matter or materials discussed in the manuscript apart from those disclosed.

## Supporting information




**Supplementary Materials**: brb371323‐sup‐0001‐Table S1‐S32.docx

## Data Availability

Data that support the findings of this study are available from the UK Medical Cannabis Registry. Restrictions apply to the availability of these data. Data specifications and applications are available from the corresponding author.
