# Supplementary material for: UK Medical Cannabis Registry: A Clinical Outcomes Analysis for Migraine
Source: Brain Behav. 2026 Apr 6;16(4):e71323. doi: 10.1002/brb3.71323 (PMC13053313; doi:10.1002/brb3.71323)
Supplement: Supplementary file 1 — Supplementary Materials: brb371323‐sup‐0001‐Table S1‐S32.docx [file BRB3-16-e71323-s001.docx]

**Supplementary Appendix**

| **Comorbidity** | **Score if present** | **Scoring Information** |
| --- | --- | --- |
| ***Myocardial Infarction*** | +1 | History of probable or definite myocardial infarction, indicated by electrocardiogram and/or enzyme changes. |
| ***Congestive Heart Failure*** | +1 | Exertional and/or paroxysmal nocturnal dyspnoea, responsive to digitalis, diuretics or medications reducing afterload. |
| ***Peripheral Vascular Disease*** | +1 | Intermittent claudication, bypass secondary to arterial deficiency, history of gangrene or acute arterial insufficiency, untreated thoracic/abdominal aneurysm measuring ≥6 cm. |
| ***Cerebrovascular Accident (CVA) or Transient Ischaemic Attack*** | +1 | History of cerebrovascular accident with minor or no residua or transient ischaemic attack. If a CVA resulted in hemiplegia, only hemiplegia should be scored. |
| ***Hemiplegia*** | +2 | Hemiplegia or paraplegia of any cause. |
| ***Dementia*** | +1 | Including patients with a chronic cognitive deficit. |
| ***Chronic Pulmonary Disease*** | +1 | Asthma, chronic bronchitis or emphysema (chronic obstructive pulmonary disorder) or chronic lung disease. Ongoing symptoms should be present e.g. dyspnoea at rest/ on exertion or cough. |
| ***Connective Tissue Disease/ Rheumatological disease*** | +1 | Including patients with Ehler Danlos syndrome, Marfans syndrome, mixed connective tissue disease, polymyositis, polymyalgia rheumatica, systemic lupus erythematous, Sjogrens syndrome, sarcoidosis or any form of systemic vasculitis. |
| ***Peptic Ulcer Disease*** | +1 | Patients who have received treatment for ulcer disease and those with a history of ulcer-related bleeding. |
| ***Moderate to severe CKD*** | +2 | Moderate – creatinine > 3mg/dL  Severe – on dialysis or post kidney transplant |
| ***Leukemia*** | +2 | Including acute and chronic lymphocytic leukemia, acute and chronic myeloid leukemia and polycythaemia vera. |
| ***Lymphoma*** | +2 | Including Hodgkins, lymphosarcoma, myeloma, Waldenstrom’s macroglobulinaemia and other lymphomas. |
| ***Acquired Immune- Deficiency Syndrome*** | +6 |  |
| ***Liver Disease*** | Mild = +1  Moderate or Severe = +3 | Mild – chronic hepatitis or cirrhosis without portal hypertension.  Moderate – cirrhosis with portal hypertension without history of variceal bleeding.  Severe – cirrhosis with portal hypertension and history of variceal bleeding. |
| ***Diabetes Mellitus*** | Uncomplicated = +1  End organ damage = +2 | End organ damage = macro or microvascular complications including retinopathy, neuropathy, or nephropathy because of diabetes. |
| ***Solid tumour*** | Localised = +2  Metastatic = +6 |  |
| ***Age*** | 50-59 = +1  60-69 = +2  70-79 = +3  ≥ 80 = +4 |  |

**Supplementary Table 1: A table showing the comorbidities, scoring and classification definitions relevant to the Charlson Comorbidity Index.** CKD – chronic kidney disease.

| **Severity** | **Description** |
| --- | --- |
| **Mild** | This means you might not even notice symptoms, or they're very mild. You don't need any medical treatment. |
| **Moderate** | This means noticeable symptoms that might require some simple treatment. You can still do most daily activities but might have trouble with complex tasks like cooking complicated meals or managing finances. |
| **Severe** | This means serious symptoms that might require going to hospital. You have trouble with basic self-care like washing yourself, getting dressed, or feeding yourself without help |
| **Life threatening/ disabling** | This means an emergency situation where you need urgent medical help to prevent death. |

**Supplementary Table 2: A table showing the descriptions that were given to patients of the different severities of adverse events when they were self-reporting.** Descriptions align with the Common Terminology of Criteria for Adverse Events 4.0.

| **Timepoint** | **HIT-6** n (%) | **MIDAS** n (%) | **EQ-5D-5L** n (%) | **GAD-7** n (%) | **SQS** n (%) | **PGIC** n (%) |
| --- | --- | --- | --- | --- | --- | --- |
| **Baseline** | 12 (5.91) | 13 (6.40) | 1 (0.49) | 0 (0.00) | 2 (0.99) |  |
| **1-month** | 32 (15.76) | 32 (15.76) | 23 (11.33) | 20 (9.85) | 24 (11.82) | 20 (9.85) |
| **3-months** | 52 (25.62) | 52 (25.62) | 44 (21.67) | 42 (20.69) | 45 (22.17) | 41 (20.20) |
| **6-months** | 74 (36.45) | 74 (36.45) | 65 (32.02) | 63 (31.03) | 67 (33.00) | 63 (31.03) |
| **12-months** | 91 (44.83) | 91 (44.83) | 84 (41.38) | 83 (40.89) | 85 (41.87) | 84 (41.38) |
| **18-months** | 106 (52.22) | 107 (52.71) | 100 (49.26) | 99 (48.77) | 102 (50.25) | 98 (48.28) |
| **24-months** | 123 (60.59) | 123 (60.59) | 119 (58.62) | 118 (58.13) | 120 (59.11) | 117 (57.64) |

**Supplementary Table 3:** **A table demonstrating the amount and proportion of missing data patient-reported outcome measures at baseline, 1-, 3-, 6-, 12-, 18- and 24-months.** HIT-6 – headache impact test 6, MIDAS – migraine disability assessment test, EQ-5D-5L – EuroQol 5-dimensions 5-levels, GAD-7 – generalised anxiety disorder 7, SQS – single item sleep quality scale, PGIC - patient global impression of change score.

| **Government Office Region** | **n (%)** |
| --- | --- |
| Channel Islands | 11 (5.4) |
| East Midlands | 3 (1.5) |
| East of England | 13 (6.4) |
| London | 28 (13.8) |
| North East | 10 (4.9) |
| North West | 19 (9.4) |
| Northern Ireland | 3 (1.5) |
| Scotland | 51 (25.1) |
| South East | 19 (9.4) |
| South West | 14 (6.9) |
| Wales | 5 (2.5) |
| West Midlands | 17 (8.4) |
| Yorkshire and the Humber | 10 (4.9) |

**Supplementary Table 4**: **A table showing the proportion of patients from each government office region of England, Wales, Scotland and the crown dependencies.**

| **Condition** | **Primary n (%)** | **Secondary n (%)** | **Tertiary n (%)** |
| --- | --- | --- | --- |
| Migraine | 203 (100.0) | 0 (0.0) | 0 (0.0) |
| Anxiety | 0 (0.0) | 20 (9.9) | 3 (1.5) |
| Attention deficit hyperactivity disorder | 0 (0.0) | 2 (1.0) | 0 (0.0) |
| Chronic non-cancer pain | 0 (0.0) | 9 (4.4) | 2 (1.0) |
| Depression | 0 (0.0) | 6 (3.0) | 3 (1.5) |
| Fibromyalgia | 0 (0.0) | 8 (3.9) | 0 (0.0) |
| Headache | 0 (0.0) | 1 (0.5) | 1 (0.5) |
| Hypermobility | 0 (0.0) | 3 (1.5) | 0 (0.0) |
| Insomnia | 0 (0.0) | 9 (4.4) | 6 (3.0) |
| Neuropathic pain | 0 (0.0) | 3 (1.5) | 0 (0.0) |
| NR | 0 (0.0) | 139 (68.5) | 182 (89.7) |
| Post Traumatic Stress Disorder (PTSD) | 0 (0.0) | 0 (0.0) | 4 (2.0) |
| Other | 0 (0.0) | 3 (1.5) | 2 (1.0) |

**Supplementary Table 5: A table showing the primary, secondary and tertiary diagnoses of patients.** A diagnosis of ‘Other’ summates diagnoses which had a frequency of 1, to maintain anonymity. NR – not recorded.

| **Variable** | **Mean ± SD / n (%)** |
| --- | --- |
| **Alcohol units per week** | 1.89 ± 4.24 |
| **Smoking Status** |  |
| Current smoker | 42 (20.7) |
| Ex-smoker | 81 (39.9) |
| Never smoked | 80 (39.4) |
| **Cannabis Status** |  |
| Current user | 119 (58.6) |
| Ex-user | 37 (18.2) |
| Never used | 47 (23.2) |

**Supplementary Table 6: A table showing the mean alcohol consumption in units per week, smoking status and cannabis use status.** SD – standard deviation.

| **Variable** | **Mean ± SD / n (%)** |
| --- | --- |
| **Cannabis Use Frequency (current users)** |  |
| <1 times per month | 2 (1.7) |
| >1 times per month | 4 (3.4) |
| 1-2 times per week | 10 (8.4) |
| Every day | 86 (72.3) |
| Every other day | 17 (14.3) |
| **Cannabis Routes (current users)** |  |
| Ingestion | 47 (39.5) |
| Smoking | 63 (52.9) |
| Vaporizing | 91 (76.5) |
| Liquid cannabis vapes | 0 (0.0) |
| Topical | 5 (4.2) |
| **Grams per day used (current users)** | 1.22 ± 1.04 |
| **Gram years** |  |
| Current Users | 13.65 ± 18.31 |
| Ex-users | 16.64 ± 30.40 |

**Supplementary Table 7: A table showing the frequency of cannabis use, route of cannabis administration and consumption in grams per day of current cannabis users**. These statistics reflect recreational cannabis use, prior to initiating medical cannabis treatment. For all current/ previous cannabis users, gram years was also measured. Gram years is a metric calculated by multiplying average daily cannabis consumption in grams by total years of consumption.

| **Combination** | **Baseline Median CBD Dose (IQR)** | **1-month CBD Dose** | **3-month CBD Dose** | **6-month CBD Dose** | **12-month CBD Dose** | **18-month CBD Dose** | **24-month CBD Dose** |
| --- | --- | --- | --- | --- | --- | --- | --- |
| Dried Flower, Oil | 21.00 (20.00-21.00) | 25.50 (20.00-25.78) | 25.50 (20.00-58.00) | 26.05 (20.00-58.55) | 35.50 (20.89-76.58) | 33.50 (21.76-80.53) | 40.65 (22.13-80.72) |
| Oils | 20.00 (20.00-20.05) | 20.54 (20.00-52.5) | 24.03 (20.00-52.50) | 34.03 (20.28-52.50) | 20.55 (20.28-51.55) | 20.55 (20.28-51.55) | 20.55 (20.21-50.56) |
| Dried Flower | 1.00 (1.00-2.00) | 5.50 (5.50-11.00) | 11.00 (5.50-22.00) | 11.00 (5.50-21.75) | 11.00 (5.50-56.34) | 11.00 (5.50-55.00) | 11.00 (5.50-17.88) |
| Capsule, Dried flower, Oil |  |  | 55.00 (55.00-55.00) | 41.00 (41.00-41.00) | 35.25 (32.38-38.13) |  |  |
| Dried flower, Oil, Pastille |  |  |  |  | 0.00 (0.00-0.00) | 35.06 (33.17-36.96) | 41.70 (38.33-50.24) |
| Dried flower/flos, Pastille |  |  |  |  |  | 22.25 (22.25-22.25) | 19.50 (18.13-29.81) |
| Capsule, Dried flower/flos |  |  |  |  |  | 41.00 (41.00-41.00) | 41.00 (41.00-41.00) |
| Dried flower/flos, Vape Cartridge |  |  |  |  |  | 18.16 (18.16-18.16) |  |
| Dried flower/flos, Oil, Vape Cartridge |  |  |  |  |  |  | 55.00 (55.00-55.00) |
| Dried flower/flos, Pastille, Vape Cartridge |  |  |  |  |  |  | 16.75 (16.75-16.75) |

**Supplementary Table 8: A table showing the CBD doses for different product combinations at baseline, 1-, 3-, 6-, 12-, 18- and 24-months** post treatment initiation. Values are medians with IQR. CBD – cannabidiol, IQR – interquartile range.

| **Combination** | **Baseline Median THC Dose (IQR)** | | **1-month Median THC Dose (IQR)** | **3-month Median THC Dose (IQR)** | **6-month Median THC Dose (IQR)** | **12-month Median THC Dose (IQR)** | **18-month Median THC Dose (IQR)** | **24-month Median THC Dose (IQR)** |
| --- | --- | --- | --- | --- | --- | --- | --- | --- |
| Dried flower/flos, Oil | | 20.00 (20.00-22.00) | 111.60 (110.00-121.00) | 121.00 (110.00–152.25) | 126.12 (112.00–219.00) | 138.52 (115.50–231.00) | 157.50 (115.50–237.25) | 134.75 (110.91–244.88) |
| Oil | | 2.00 (1.00-2.60) | 6.55 (5.50-11.00) | 9.50 (5.62–12.30) | 10.25 (6.00–13.18) | 10.00 (5.75–12.50) | 10.00 (5.75–13.00) | 10.75 (5.88–13.00) |
| Dried flower/flos | | 20.00 (19.00-26.00) | 110.00 (108.63-204.88) | 192.50 (110.00–220.00) | 209.00 (110.00–255.75) | 209.00 (110.00–265.62) | 209.00 (115.50–275.00) | 217.25 (115.50–281.19) |
| Capsule, Dried flower, Oil | |  |  | 136.00 (136.00–136.00) |  |  |  |  |
| Capsule, Dried flower/flos | |  |  |  | 235.00 (235.00–235.00) | 331.12 (283.06–379.19) | 235.00 (235.00–235.00) | 235.00 (235.00–235.00) |
| Dried flower/flos, Oil, Pastille | |  |  |  |  | 128.62 (128.62–128.62) | 204.68 (192.71–216.64) | 159.00 (133.75–199.31) |
| Dried flower/flos, Pastille | |  |  |  |  |  | 267.00 (265.62–268.38) | 209.25 (176.25–245.00) |
| Dried flower/flos, Vape Cartridge | |  |  |  |  |  | 363.20 (363.20–363.20) | 364.86 (284.95–452.28) |
| Dried flower/flos, Oil, Vape Cartridge | |  |  |  |  |  |  | 293.36 (293.36–293.36) |
| Dried flower/flos, Pastille, Vape Cartridge | |  |  |  |  |  |  | 155.30 (155.30–155.30) |

**Supplementary Table 9: A table showing the THC doses for different product combinations at baseline, 1-, 3-, 6-, 12-, 18- and 24-months** post treatment initiation. Values are medians with IQR. THC – tetrahydrocannabinol, IQR – interquartile range.

| **HIT-6 Comparison** | **Mean  Difference** | **SD of  Difference** | **Formatted p-value** | **Significant** |
| --- | --- | --- | --- | --- |
| Baseline vs 1 month | 5.192 | 7.346 | <0.001 | Yes |
| Baseline vs 3 months | 7.586 | 9.296 | <0.001 | Yes |
| Baseline vs 6 months | 8.158 | 9.277 | <0.001 | Yes |
| Baseline vs 12 months | 8.911 | 10.275 | <0.001 | Yes |
| Baseline vs 18 months | 4.468 | 10.661 | <0.001 | Yes |
| Baseline vs 24 months | 6.286 | 13.602 | <0.001 | Yes |
| 1 month vs 3 months | 2.394 | 9.052 | 0.005 | Yes |
| 1 month vs 6 months | 2.966 | 9.541 | <0.001 | Yes |
| 1 month vs 12 months | 3.719 | 10.061 | <0.001 | Yes |
| 1 month vs 18 months | -0.724 | 10.641 | 1.000 | No |
| 1 month vs 24 months | 1.094 | 15.348 | 1.000 | No |
| 3 months vs 6 months | 0.571 | 8.850 | 1.000 | No |
| 3 months vs 12 months | 1.325 | 10.324 | 1.000 | No |
| 3 months vs 18 months | -3.118 | 10.638 | <0.001 | Yes |
| 3 months vs 24 months | -1.300 | 16.320 | 1.000 | No |
| 6 months vs 12 months | 0.754 | 10.110 | 1.000 | No |
| 6 months vs 18 months | -3.690 | 11.492 | <0.001 | Yes |
| 6 months vs 24 months | -1.872 | 16.200 | 1.000 | No |
| 12 months vs 18 months | -4.443 | 12.980 | <0.001 | Yes |
| 12 months vs 24 months | -2.626 | 16.222 | 0.464 | No |
| 18 months vs 24 months | 1.818 | 16.617 | 1.000 | No |

**Supplementary Table 10: A table showing pairwise comparisons between HIT-6 scores at different timepoints amongst patients**, including the mean of differences between scores, the standard deviation, p-values for this difference and whether findings reached statistical significance. HIT-6 – headache impact test 6. SD – standard deviation.

| **MIDAS Comparison** | **Mean  Difference** | **SD of  Difference** | **Formatted  p-value** | **Significant** |
| --- | --- | --- | --- | --- |
| Baseline vs 1 month | 33.433 | 116.190 | 0.001 | Yes |
| Baseline vs 3 months | 46.675 | 128.130 | <0.001 | Yes |
| Baseline vs 6 months | 29.901 | 135.115 | 0.039 | Yes |
| Baseline vs 12 months | 40.084 | 139.457 | 0.001 | Yes |
| Baseline vs 18 months | 4.507 | 140.178 | 1 | No |
| Baseline vs 24 months | 19.813 | 158.493 | 1 | No |
| 1 month vs 3 months | 13.241 | 99.533 | 1 | No |
| 1 month vs 6 months | -3.532 | 118.065 | 1 | No |
| 1 month vs 12 months | 6.650 | 104.174 | 1 | No |
| 1 month vs 18 months | -28.926 | 128.154 | 0.032 | Yes |
| 1 month vs 24 months | -13.621 | 136.497 | 1 | No |
| 3 months vs 6 months | -16.773 | 120.432 | 1 | No |
| 3 months vs 12 months | -6.591 | 111.886 | 1 | No |
| 3 months vs 18 months | -42.167 | 122.645 | <0.001 | Yes |
| 3 months vs 24 months | -26.862 | 136.206 | 0.114 | No |
| 6 months vs 12 months | 10.182 | 83.717 | 1 | No |
| 6 months vs 18 months | -25.394 | 157.300 | 0.472 | No |
| 6 months vs 24 months | -10.089 | 166.891 | 1 | No |
| 12 months vs 18 months | -35.576 | 143.033 | 0.01 | Yes |
| 12 months vs 24 months | -20.271 | 151.488 | 1 | No |
| 18 months vs 24 months | 15.305 | 167.674 | 1 | No |

**Supplementary Table 11: A table showing pairwise comparisons between MIDAS scores at different timepoints amongst patients**, including the mean of differences between scores, the standard deviation, p-values for this difference and whether findings reached statistical significance. MIDAS - Migraine Disability Assessment Test. SD – standard deviation.

| **EQ-5D-5L Index Score Comparison** | **Mean  Difference** | **SD of  Difference** | **Formatted  p-value** | **Significant** |
| --- | --- | --- | --- | --- |
| Baseline vs 1 month | -0.138 | 0.237 | <0.001 | Yes |
| Baseline vs 3 months | -0.122 | 0.297 | <0.001 | Yes |
| Baseline vs 6 months | -0.143 | 0.271 | <0.001 | Yes |
| Baseline vs 12 months | -0.120 | 0.277 | <0.001 | Yes |
| Baseline vs 18 months | -0.125 | 0.293 | <0.001 | Yes |
| Baseline vs 24 months | -0.138 | 0.315 | <0.001 | Yes |
| 1 month vs 3 months | 0.016 | 0.255 | 0.016 | No |
| 1 month vs 6 months | -0.004 | 0.233 | <0.001 | No |
| 1 month vs 12 months | 0.018 | 0.252 | 0.018 | No |
| 1 month vs 18 months | 0.014 | 0.291 | 0.014 | No |
| 1 month vs 24 months | 0.000 | 0.359 | <0.001 | No |
| 3 months vs 6 months | -0.020 | 0.245 | <0.001 | No |
| 3 months vs 12 months | 0.002 | 0.264 | 0.002 | No |
| 3 months vs 18 months | -0.002 | 0.288 | <0.001 | No |
| 3 months vs 24 months | -0.016 | 0.318 | <0.001 | No |
| 6 months vs 12 months | 0.023 | 0.202 | 0.023 | No |
| 6 months vs 18 months | 0.018 | 0.257 | 0.018 | No |
| 6 months vs 24 months | 0.005 | 0.297 | 0.005 | No |
| 12 months vs 18 months | -0.005 | 0.268 | <0.001 | No |
| 12 months vs 24 months | -0.018 | 0.260 | <0.001 | No |
| 18 months vs 24 months | -0.013 | 0.298 | <0.001 | No |

**Supplementary Table 12: A table showing pairwise comparisons between EQ-5D-5L scores at different timepoints amongst patients**, including the mean of differences between scores, the standard deviation, p-values for this difference and whether findings reached statistical significance. EQ-5D-5L – EuroQol 5-Dimensions 5-Levels, SD – standard deviation.

| **GAD-7 Score Comparison** | **Mean  Difference** | **SD of  Difference** | **Formatted  p-value** | **Significant** |
| --- | --- | --- | --- | --- |
| Baseline vs 1 month | 2.631 | 5.202 | 2.631 | Yes |
| Baseline vs 3 months | 2.330 | 5.459 | 2.330 | Yes |
| Baseline vs 6 months | 1.828 | 5.739 | 1.828 | Yes |
| Baseline vs 12 months | 1.882 | 7.011 | 1.882 | Yes |
| Baseline vs 18 months | 2.241 | 6.091 | 2.241 | Yes |
| Baseline vs 24 months | 2.271 | 7.710 | 2.271 | Yes |
| 1 month vs 3 months | -0.300 | 4.143 | <0.001 | No |
| 1 month vs 6 months | -0.803 | 4.696 | <0.001 | No |
| 1 month vs 12 months | -0.749 | 5.846 | <0.001 | No |
| 1 month vs 18 months | -0.389 | 5.008 | <0.001 | No |
| 1 month vs 24 months | -0.360 | 6.628 | <0.001 | No |
| 3 months vs 6 months | -0.502 | 4.149 | <0.001 | No |
| 3 months vs 12 months | -0.448 | 5.513 | <0.001 | No |
| 3 months vs 18 months | -0.089 | 5.590 | <0.001 | No |
| 3 months vs 24 months | -0.059 | 6.220 | <0.001 | No |
| 6 months vs 12 months | 0.054 | 5.407 | 0.054 | No |
| 6 months vs 18 months | 0.414 | 5.261 | 0.414 | No |
| 6 months vs 24 months | 0.443 | 6.204 | 0.443 | No |
| 12 months vs 18 months | 0.360 | 4.954 | 0.360 | No |
| 12 months vs 24 months | 0.389 | 5.787 | 0.389 | No |
| 18 months vs 24 months | 0.030 | 6.059 | 0.030 | No |

**Supplementary Table 13: A table showing pairwise comparisons between GAD-7 scores at different timepoints amongst patients**, including the mean of differences between scores, the standard deviation, p-values for this difference and whether findings reached statistical significance. GAD-7 – Generalised anxiety disorder 7, SD – standard deviation.

| **SQS Score Comparison** | **Mean  Difference** | **SD of  Difference** | **Formatted  p-value** | **Significant** |
| --- | --- | --- | --- | --- |
| Baseline vs 1 month | -1.537 | 2.545 | <0.001 | Yes |
| Baseline vs 3 months | -1.675 | 2.874 | <0.001 | Yes |
| Baseline vs 6 months | -1.404 | 3.038 | <0.001 | Yes |
| Baseline vs 12 months | -1.709 | 3.003 | <0.001 | Yes |
| Baseline vs 18 months | -1.990 | 3.094 | <0.001 | Yes |
| Baseline vs 24 months | -2.123 | 3.935 | <0.001 | Yes |
| 1 month vs 3 months | -0.138 | 2.659 | 1.000 | No |
| 1 month vs 6 months | 0.133 | 2.604 | 1.000 | No |
| 1 month vs 12 months | -0.172 | 2.810 | 1.000 | No |
| 1 month vs 18 months | -0.453 | 3.083 | 0.787 | No |
| 1 month vs 24 months | -0.586 | 3.762 | 0.578 | No |
| 3 months vs 6 months | 0.271 | 2.616 | 1.000 | No |
| 3 months vs 12 months | -0.034 | 2.881 | 1.000 | No |
| 3 months vs 18 months | -0.315 | 2.840 | 1.000 | No |
| 3 months vs 24 months | -0.448 | 3.573 | 1.000 | No |
| 6 months vs 12 months | -0.305 | 2.868 | 1.000 | No |
| 6 months vs 18 months | -0.586 | 3.241 | 0.224 | No |
| 6 months vs 24 months | -0.719 | 3.446 | 0.069 | No |
| 12 months vs 18 months | -0.281 | 2.973 | 1.000 | No |
| 12 months vs 24 months | -0.414 | 3.695 | 1.000 | No |
| 18 months vs 24 months | -0.133 | 3.425 | 1.000 | No |

**Supplementary Table 14: A table showing pairwise comparisons between SQS scores at different timepoints amongst patients**, including the mean of differences between scores, the standard deviation, p-values for this difference and whether findings reached statistical significance. SQS – Single Item Sleep Quality Scale, SD – standard deviation.

| **EQ-5D-5L Mobility Comparison** | **Mean  Difference** | **SD of  Difference** | **Formatted  p-value** | **Significant** |
| --- | --- | --- | --- | --- |
| Baseline vs 1 month | 0.089 | 0.590 | 0.705 | No |
| Baseline vs 3 months | 0.015 | 0.920 | 1.000 | No |
| Baseline vs 6 months | -0.010 | 0.912 | 1.000 | No |
| Baseline vs 12 months | -0.222 | 1.022 | 0.048 | Yes |
| Baseline vs 18 months | -0.039 | 0.943 | 1.000 | No |
| Baseline vs 24 months | -0.512 | 1.433 | <0.001 | Yes |
| 1 month vs 3 months | -0.074 | 0.895 | 1.000 | No |
| 1 month vs 6 months | -0.099 | 0.923 | 1.000 | No |
| 1 month vs 12 months | -0.310 | 0.968 | <0.001 | Yes |
| 1 month vs 18 months | -0.128 | 0.987 | 1.000 | No |
| 1 month vs 24 months | -0.601 | 1.443 | <0.001 | Yes |
| 3 months vs 6 months | -0.025 | 0.870 | 1.000 | No |
| 3 months vs 12 months | -0.236 | 0.881 | 0.004 | Yes |
| 3 months vs 18 months | -0.054 | 0.778 | 1.000 | No |
| 3 months vs 24 months | -0.527 | 1.195 | <0.001 | Yes |
| 6 months vs 12 months | -0.212 | 0.808 | 0.005 | Yes |
| 6 months vs 18 months | -0.030 | 0.969 | 1.000 | No |
| 6 months vs 24 months | -0.502 | 1.248 | <0.001 | Yes |
| 12 months vs 18 months | 0.182 | 0.970 | 0.169 | No |
| 12 months vs 24 months | -0.291 | 1.222 | 0.018 | Yes |
| 18 months vs 24 months | -0.473 | 1.045 | <0.001 | Yes |

**Supplementary Table 15: A table showing pairwise comparisons between EQ-5D-5L Mobility scores at different timepoints amongst patients**, including the mean of differences between scores, the standard deviation, p-values for this difference and whether findings reached statistical significance. A higher score represents improvement. EQ-5D-5L – EuroQol 5-dimension 5-levels, SD – standard deviation.

| **EQ-5D-5L Selfcare Comparison** | **Mean  Difference** | **SD of  Difference** | **Formatted  p-value** | **Significant** |
| --- | --- | --- | --- | --- |
| Baseline vs 1 month | 0.034 | 0.608 | 1.000 | No |
| Baseline vs 3 months | -0.020 | 0.751 | 1.000 | No |
| Baseline vs 6 months | -0.103 | 0.786 | 1.000 | No |
| Baseline vs 12 months | -0.103 | 0.847 | 1.000 | No |
| Baseline vs 18 months | -0.335 | 1.013 | <0.001 | Yes |
| Baseline vs 24 months | -0.261 | 0.968 | 0.003 | Yes |
| 1 month vs 3 months | -0.054 | 0.753 | 1.000 | No |
| 1 month vs 6 months | -0.138 | 0.803 | 0.319 | No |
| 1 month vs 12 months | -0.138 | 0.683 | 0.093 | No |
| 1 month vs 18 months | -0.369 | 0.905 | <0.001 | Yes |
| 1 month vs 24 months | -0.296 | 1.044 | 0.002 | Yes |
| 3 months vs 6 months | -0.084 | 0.795 | 1.000 | No |
| 3 months vs 12 months | -0.084 | 0.855 | 1.000 | No |
| 3 months vs 18 months | -0.315 | 0.980 | <0.001 | Yes |
| 3 months vs 24 months | -0.241 | 0.947 | 0.008 | Yes |
| 6 months vs 12 months | 0.000 | 0.827 | 1.000 | No |
| 6 months vs 18 months | -0.232 | 0.833 | 0.002 | Yes |
| 6 months vs 24 months | -0.158 | 0.904 | 0.289 | No |
| 12 months vs 18 months | -0.232 | 0.675 | <0.001 | Yes |
| 12 months vs 24 months | -0.158 | 1.012 | 0.580 | No |
| 18 months vs 24 months | 0.074 | 1.043 | 1.000 | No |

**Supplementary Table 16: A table showing pairwise comparisons between EQ-5D-5L Selfcare scores at different timepoints amongst patients**, including the mean of differences between scores, the standard deviation, p-values for this difference and whether findings reached statistical significance. A higher score represents improvement. EQ-5D-5L – EuroQol 5-dimension 5-levels, SD – standard deviation.

| **EQ-5D-5L Usual Activities Comparison** | **Mean  Difference** | **SD of  Difference** | **Formatted  p-value** | **Significant** |
| --- | --- | --- | --- | --- |
| Baseline vs 1 month | 0.345 | 0.964 | <0.001 | Yes |
| Baseline vs 3 months | 0.207 | 1.150 | 0.234 | No |
| Baseline vs 6 months | 0.241 | 1.296 | 0.180 | No |
| Baseline vs 12 months | 0.118 | 1.352 | 1.000 | No |
| Baseline vs 18 months | 0.103 | 1.540 | 1.000 | No |
| Baseline vs 24 months | -0.030 | 1.476 | 1.000 | No |
| 1 month vs 3 months | -0.138 | 0.995 | 1.000 | No |
| 1 month vs 6 months | -0.103 | 1.216 | 1.000 | No |
| 1 month vs 12 months | -0.227 | 1.176 | 0.139 | No |
| 1 month vs 18 months | -0.241 | 1.478 | 0.440 | No |
| 1 month vs 24 months | -0.374 | 1.488 | 0.009 | Yes |
| 3 months vs 6 months | 0.034 | 0.997 | 1.000 | No |
| 3 months vs 12 months | -0.089 | 1.157 | 1.000 | No |
| 3 months vs 18 months | -0.103 | 1.260 | 1.000 | No |
| 3 months vs 24 months | -0.236 | 1.477 | 0.495 | No |
| 6 months vs 12 months | -0.123 | 1.194 | 1.000 | No |
| 6 months vs 18 months | -0.138 | 1.182 | 1.000 | No |
| 6 months vs 24 months | -0.271 | 1.375 | 0.115 | No |
| 12 months vs 18 months | -0.015 | 1.162 | 1.000 | No |
| 12 months vs 24 months | -0.148 | 1.338 | 1.000 | No |
| 18 months vs 24 months | -0.133 | 1.155 | 1.000 | No |

**Supplementary Table 17: A table showing pairwise comparisons between EQ-5D-5L Usual Activities scores at different timepoints amongst patients,** including the mean of differences between scores, the standard deviation, p-values for this difference and whether findings reached statistical significance. A higher score represents improvement. EQ-5D-5L – EuroQol 5-dimension 5-levels, SD – standard deviation.

| **EQ-5D-5L Pain and Discomfort Comparison** | **Mean  Difference** | **SD of  Difference** | **Formatted  p-value** | **Significant** |
| --- | --- | --- | --- | --- |
| Baseline vs 1 month | 0.680 | 1.086 | <0.001 | Yes |
| Baseline vs 3 months | 0.695 | 1.373 | <0.001 | Yes |
| Baseline vs 6 months | 0.823 | 1.197 | <0.001 | Yes |
| Baseline vs 12 months | 0.704 | 1.174 | <0.001 | Yes |
| Baseline vs 18 months | 0.719 | 1.229 | 1.000 | No |
| Baseline vs 24 months | 0.749 | 1.529 | 1.000 | No |
| 1 month vs 3 months | 0.015 | 1.137 | 1.000 | No |
| 1 month vs 6 months | 0.143 | 1.087 | 1.000 | No |
| 1 month vs 12 months | 0.025 | 1.224 | 1.000 | No |
| 1 month vs 18 months | 0.039 | 1.238 | 0.024 | Yes |
| 1 month vs 24 months | 0.069 | 1.658 | 0.102 | No |
| 3 months vs 6 months | 0.128 | 1.123 | 1.000 | No |
| 3 months vs 12 months | 0.010 | 1.286 | 1.000 | No |
| 3 months vs 18 months | 0.025 | 1.288 | 0.142 | No |
| 3 months vs 24 months | 0.054 | 1.466 | 0.143 | No |
| 6 months vs 12 months | -0.118 | 1.097 | 1.000 | No |
| 6 months vs 18 months | -0.103 | 1.212 | 0.090 | No |
| 6 months vs 24 months | -0.074 | 1.525 | 0.320 | No |
| 12 months vs 18 months | 0.015 | 1.261 | 0.306 | No |
| 12 months vs 24 months | 0.044 | 1.500 | 0.401 | No |
| 18 months vs 24 months | 0.030 | 1.410 | 1.000 | No |

**Supplementary Table 18: A table showing pairwise comparisons between EQ-5D-5L Pain and Discomfort scores at different timepoints amongst patients**, including the mean of differences between scores, the standard deviation, p-values for this difference and whether findings reached statistical significance. A higher score represents improvement. EQ-5D-5L – EuroQol 5-dimension 5-levels, SD – standard deviation.

| **EQ-5D-5L Anxiety and Depression Comparison** | **Mean  Difference** | **SD of  Difference** | **Formatted  p-value** | **Significant** |
| --- | --- | --- | --- | --- |
| Baseline vs 1 month | 0.424 | 0.878 | <0.001 | Yes |
| Baseline vs 3 months | 0.409 | 1.192 | <0.001 | Yes |
| Baseline vs 6 months | 0.394 | 1.144 | <0.001 | Yes |
| Baseline vs 12 months | 0.379 | 1.202 | <0.001 | Yes |
| Baseline vs 18 months | 0.148 | 1.403 | 1.000 | No |
| Baseline vs 24 months | 0.158 | 1.454 | 1.000 | No |
| 1 month vs 3 months | -0.015 | 0.957 | 1.000 | No |
| 1 month vs 6 months | -0.030 | 0.933 | 1.000 | No |
| 1 month vs 12 months | -0.044 | 1.073 | 1.000 | No |
| 1 month vs 18 months | -0.276 | 1.191 | 0.024 | Yes |
| 1 month vs 24 months | -0.266 | 1.331 | 0.102 | No |
| 3 months vs 6 months | -0.015 | 1.101 | 1.000 | No |
| 3 months vs 12 months | -0.030 | 1.112 | 1.000 | No |
| 3 months vs 18 months | -0.261 | 1.359 | 0.142 | No |
| 3 months vs 24 months | -0.251 | 1.309 | 0.143 | No |
| 6 months vs 12 months | -0.015 | 1.074 | 1.000 | No |
| 6 months vs 18 months | -0.246 | 1.214 | 0.090 | No |
| 6 months vs 24 months | -0.236 | 1.376 | 0.320 | No |
| 12 months vs 18 months | -0.232 | 1.339 | 0.306 | No |
| 12 months vs 24 months | -0.222 | 1.337 | 0.401 | No |
| 18 months vs 24 months | 0.010 | 1.130 | 1.000 | No |

**Supplementary Table 19: A table showing pairwise comparisons between EQ-5D-5L Anxiety and Depression scores at different timepoints amongst patients**, including the mean of differences between scores, the standard deviation, p-values for this difference and whether findings reached statistical significance. A higher score represents improvement. EQ-5D-5L – EuroQol 5-dimension 5-levels, SD – standard deviation.

| **Variable** | **Odds Ratio (95% CI)** | **p-value** |
| --- | --- | --- |
| **Age** |  |  |
| Under 30 (reference) | 1.00 |  |
| 31-40 | 0.86 (0.40-1.86) | 0.709 |
| 41-50 | 1.00 (0.45-2.20) | 1.000 |
| 51-60 | 0.50 (0.14-1.64) | 0.258 |
| Over 60 | 0.75 (0.20-2.77) | 0.661 |
| **Sex** |  |  |
| Male (reference) | 1.00 |  |
| Female | 0.84 (0.48-1.46) | 0.527 |
| **BMI** |  |  |
| 20-24.99 (reference) | 1.00 |  |
| Under 20 | 0.92 (0.33-2.61) | 0.875 |
| 25-29.99 | 1.61 (0.77-3.42) | 0.205 |
| 30-34.99 | 0.83 (0.35-1.98) | 0.671 |
| 35+ | 0.57 (0.22-1.41) | 0.226 |
| **Cannabis Status** |  |  |
| Never Used (reference) | 1.00 |  |
| Current User | 1.80 (0.91-3.60) | 0.092 |
| Previous User | 1.98 (0.83-4.82) | 0.126 |
| **24-month CBMP Use** |  |  |
| Oil (reference) | 1.00 |  |
| Dried flowers, Oils | 2.28 (1.01-5.35) | 0.051 |
| Dried flowers | 2.01 (0.85-4.90) | 0.115 |
| Other | 3.06 (0.92-10.98) | 0.074 |
| **24-month CBD Dose** |  |  |
| Minimum to Q1 | 1.00 |  |
| Q1 to Median | 0.83 (0.38-1.80) | 0.646 |
| Median to Q3 | 0.50 (0.22-1.11) | 0.090 |
| Q3 to Maximum | 1.36 (0.62-3.02) | 0.444 |
| **24-month THC Dose** |  |  |
| Minimum to Q1 | 1.00 |  |
| Q1 to Median | 1.95 (0.90-4.31) | 0.095 |
| Median to Q3 | 2.07 (0.94-4.65) | 0.073 |
| Q3 to Maximum | 2.04 (0.93-4.54) | 0.076 |
| **Baseline SQS Score** |  |  |
| Good-Excellent: 7-10 (reference) | 1 |  |
| Fair: 4-6 | 0.77 (0.38-1.54) | 0.464 |
| Terrible - Poor: 0-3 | 0.67 (0.32-1.37) | 0.272 |
| **Baseline GAD-7 Score** |  |  |
| Less than 5 (reference) | 1 |  |
| 5-9 | 0.71 (0.35-1.43) | 0.338 |
| 10-14 | 1.96 (0.84-4.84) | 0.128 |
| 15+ | 1.00 (0.46-2.17) | 0.992 |

**Supplementary Table 20: Univariable regression results for achieving a MCID in the HIT-6 at 24-months.** MCID – minimal clinically important difference, HIT-6 – headache impact test 6, CI – confidence interval, BMI – body mass index, CBMP – cannabis-based medicinal product, CBD – cannabidiol, THC – tetrahydrocannabinol, SQS – single item sleep quality scale, GAD-7- generalised anxiety disorder 7. GAD-7 Scoring: less than 5 – no anxiety symptoms, 5-9 – mild anxiety symptoms, 10-14 – moderate anxiety symptoms, 15+ - severe anxiety symptoms.

| **Variable** | **Odds Ratio (95% CI)** | **p-value** |
| --- | --- | --- |
| **Age** |  |  |
| Under 30 (reference) | 1.00 |  |
| 31-40 | 0.89 (0.34-2.28) | 0.810 |
| 41-50 | 0.96 (0.37-2.46) | 0.927 |
| 51-60 | 0.40 (0.10-1.51) | 0.182 |
| Over 60 | 0.46 (0.10-2.17) | 0.325 |
| **Sex** |  |  |
| Male (reference) | 1.00 |  |
| Female | 1.18 (0.58-2.44) | 0.650 |
| **BMI** |  |  |
| 20-24.99 (reference) | 1.00 |  |
| Under 20 | 0.89 (0.26-3.11) | 0.853 |
| 25-29.99 | 1.32 (0.56-3.10) | 0.527 |
| 30-34.99 | 0.77 (0.27-2.15) | 0.618 |
| 35+ | 0.41 (0.14-1.15) | 0.095 |
| **Cannabis Status** |  |  |
| Never Used (reference) | 1.00 |  |
| Current User | 1.63 (0.65-4.15) | 0.295 |
| Previous User | 1.96 (0.69-5.70) | 0.212 |
| **24-month CBMP Use** |  |  |
| Oil (reference) | 1.00 |  |
| Dried flowers, Oils | 0.92 (0.22-3.87) | 0.906 |
| Dried flowers | 0.43 (0.08-2.18) | 0.318 |
| Other | 1.62 (0.27-10.33) | 0.600 |
| **24-month CBD Dose** |  |  |
| Minimum to Q1 (reference) | 1.00 |  |
| Q1 to Median | 0.47 (0.17-1.30) | 0.151 |
| Median to Q3 | 0.25 (0.08-0.75) | 0.015 |
| Q3 to Maximum | 0.89 (0.31-2.52) | 0.819 |
| **24-month THC Dose** |  |  |
| Minimum to Q1 (reference) | 1.00 |  |
| Q1 to Median | 2.11 (0.57-8.11) | 0.266 |
| Median to Q3 | 2.14 (0.58-8.00) | 0.251 |
| Q3 to Maximum | 2.00 (0.55-7.51) | 0.298 |
| **Baseline SQS Score** |  |  |
| Good-Excellent: 7-10 (reference) | 1.00 |  |
| Fair: 4-6 | 0.58 (0.24-1.38) | 0.220 |
| Terrible - Poor: 0-3 | 0.56 (0.23-1.35) | 0.200 |
| **Baseline GAD-7 Score** | |  |
| Less than 5 (reference) | 1.00 |  |
| 5-9 | 0.95 (0.42-2.16) | 0.898 |
| 10-14 | 2.77 (0.99-8.29) | 0.058 |
| 15+ | 1.34 (0.49-3.69) | 0.570 |

**Supplementary Table 21: Multivariable regression results for achieving a MCID in the HIT-6 at 24-months.** MCID – minimal clinically important difference, HIT-6 – headache impact test 6, CI – confidence interval, BMI – body mass index, CBMP – cannabis-based medicinal product, CBD – cannabidiol, THC – tetrahydrocannabinol, SQS – single item sleep quality scale, GAD-7- generalised anxiety disorder 7. GAD-7 Scoring: less than 5 – no anxiety symptoms, 5-9 – mild anxiety symptoms, 10-14 – moderate anxiety symptoms, 15+ - severe anxiety symptoms.

| **Variable** | **Odds Ratio (95% CI)** | **p-value** |
| --- | --- | --- |
| **Age** |  |  |
| Under 30 (reference) | 1.00 |  |
| 31-40 | 1.39 (0.65-3.02) | 0.395 |
| 41-50 | 1.72 (0.79-3.82) | 0.176 |
| 51-60 | 1.82 (0.55-6.30) | 0.329 |
| Over 60 | 0.86 (0.22-3.15) | 0.826 |
| **Sex** |  |  |
| Male (reference) | 1.00 |  |
| Female | 0.47 (0.26-0.82) | 0.008 |
| **BMI** |  |  |
| 20-24.99 (reference) | 1.00 |  |
| Under 20 | 1.09 (0.38-3.05) | 0.875 |
| 25-29.99 | 1.61 (0.78-3.34) | 0.197 |
| 30-34.99 | 2.08 (0.87-5.21) | 0.106 |
| 35+ | 1.76 (0.71-4.46) | 0.226 |
| **Cannabis Status** |  |  |
| Never Used (reference) | 1.00 |  |
| Current User | 1.49 (0.76-2.96) | 0.249 |
| Previous User | 1.82 (0.76-4.41) | 0.180 |
| **24-month CBMP Use** |  |  |
| Oil (reference) | 1.00 |  |
| Dried flowers, Oils | 1.67 (0.74-3.89) | 0.226 |
| Dried flowers | 2.78 (1.17-6.83) | 0.022 |
| Other | 3.06 (0.92-10.98) | 0.074 |
| **24-month CBD Dose** |  |  |
| Minimum to Q1 (reference) | 1.00 |  |
| Q1 to Median | 0.83 (0.38-1.79) | 0.639 |
| Median to Q3 | 1.08 (0.49-2.39) | 0.848 |
| Q3 to Maximum | 0.77 (0.35-1.66) | 0.503 |
| **24-month THC Dose** |  |  |
| Minimum to Q1 (reference) | 1.00 |  |
| Q1 to Median | 2.95 (1.34-6.71) | 0.008 |
| Median to Q3 | 2.45 (1.10-5.60) | 0.030 |
| Q3 to Maximum | 3.67 (1.64-8.48) | 0.002 |
| **Baseline SQS Score** |  |  |
| Good-Excellent: 7-10 (reference) | 1.00 |  |
| Fair: 4-6 | 1.46 (0.74-2.92) | 0.277 |
| Terrible - Poor: 0-3 | 1.04 (0.51-2.13) | 0.921 |
| **Baseline GAD-7 Score** |  |  |
| Less than 5 (reference) | 1.00 |  |
| 5-9 | 0.56 (0.28-1.13) | 0.108 |
| 10-14 | 1.67 (0.71-4.13) | 0.247 |
| 15+ | 0.56 (0.25-1.22) | 0.147 |

**Supplementary Table 22: Univariable regression results for achieving a MCID in the MIDAS at 24-months.** MCID – minimal clinically important difference, MIDAS – migraine disability assessment test, CI – confidence interval, BMI – body mass index, CBMP – cannabis-based medicinal product, CBD – cannabidiol, THC – tetrahydrocannabinol, SQS – single item sleep quality scale, GAD-7- generalised anxiety disorder 7. GAD-7 Scoring: less than 5 – no anxiety symptoms, 5-9 – mild anxiety symptoms, 10-14 – moderate anxiety symptoms, 15+ - severe anxiety symptoms.

| **Variable** | **Odds Ratio (95% CI)** | **p-value** |
| --- | --- | --- |
| **Age** |  |  |
| Under 30 (reference) | 1.00 |  |
| 31-40 | 1.62 (0.64-4.17) | 0.312 |
| 41-50 | 2.19 (0.87-5.66) | 0.099 |
| 51-60 | 3.09 (0.79-13.33) | 0.114 |
| Over 60 | 1.29 (0.25-6.28) | 0.751 |
| **Sex** |  |  |
| Male (reference) | 1.00 |  |
| Female | 0.48 (0.23-0.98) | 0.046 |
| **BMI** |  |  |
| 20-24.99 (reference) | 1.00 |  |
| Under 20 | 1.26 (0.37-4.37) | 0.709 |
| 25-29.99 | 0.97 (0.41-2.29) | 0.946 |
| 30-34.99 | 1.62 (0.58-4.66) | 0.363 |
| 35+ | 1.78 (0.61-5.32) | 0.293 |
| **Cannabis Status** |  |  |
| Never Used (reference) | 1.00 |  |
| Current User | 0.97 (0.38-2.42) | 0.940 |
| Previous User | 1.41 (0.49-4.08) | 0.522 |
| **24-month CBMP Use** |  |  |
| Oil (reference) | 1.00 |  |
| Dried flowers, Oils | 0.75 (0.16-3.30) | 0.710 |
| Dried flowers | 1.17 (0.21-6.31) | 0.852 |
| Other | 1.32 (0.21-8.52) | 0.765 |
| **24-month CBD Dose** |  |  |
| Minimum to Q1 (reference) | 1.00 |  |
| Q1 to Median | 1.07 (0.38-3.09) | 0.898 |
| Median to Q3 | 1.74 (0.58-5.35) | 0.323 |
| Q3 to Maximum | 0.90 (0.31-2.65) | 0.851 |
| **24-month THC Dose** |  |  |
| Minimum to Q1 (reference) | 1.00 |  |
| Q1 to Median | 3.83 (1.00-16.67) | 0.058 |
| Median to Q3 | 2.70 (0.72-11.39) | 0.154 |
| Q3 to Maximum | 4.03 (1.07-17.31) | 0.047 |
| **Baseline SQS Score** |  |  |
| Good-Excellent: 7-10 (reference) | 1.00 |  |
| Fair: 4-6 | 1.42 (0.58-3.45) | 0.440 |
| Terrible - Poor: 0-3 | 1.74 (0.72-4.32) | 0.221 |
| **Baseline GAD-7 Score** |  |  |
| Less than 5 (reference) | 1.00 |  |
| 5-9 | 0.61 (0.27-1.40) | 0.249 |
| 10-14 | 1.94 (0.70-5.67) | 0.212 |
| 15+ | 0.57 (0.21-1.58) | 0.283 |

**Supplementary Table 23: Multivariable regression results for achieving a MCID in the MIDAS at 24-months.** MCID – minimal clinically important difference, MIDAS – migraine disability assessment test, CI – confidence interval, BMI – body mass index, CBMP – cannabis-based medicinal product, CBD – cannabidiol, THC – tetrahydrocannabinol, SQS – single item sleep quality scale, GAD-7- generalised anxiety disorder 7. GAD-7 Scoring: less than 5 – no anxiety symptoms, 5-9 – mild anxiety symptoms, 10-14 – moderate anxiety symptoms, 15+ - severe anxiety symptoms.

| **Variable** | **Odds Ratio (95% CI)** | **p-value** |
| --- | --- | --- |
| **Age** |  |  |
| Under 30 (reference) | 1.00 |  |
| 31-40 | 0.98 (0.43-2.19) | 0.959 |
| 41-50 | 0.93 (0.40-2.12) | 0.867 |
| 51-60 | 1.37 (0.39-5.67) | 0.634 |
| Over 60 | 1.50 (0.38-7.56) | 0.585 |
| **Sex** |  |  |
| Male (reference) | 1.00 |  |
| Female | 0.79 (0.44-1.42) | 0.430 |
| **BMI** |  |  |
| 20-24.99 (reference) | 1.00 |  |
| Under 20 | 0.90 (0.31-2.71) | 0.843 |
| 25-29.99 | 1.92 (0.86-4.47) | 0.119 |
| 30-34.99 | 0.90 (0.37-2.28) | 0.828 |
| 35+ | 0.65 (0.26-1.65) | 0.366 |
| **Cannabis Status** |  |  |
| Never Used (reference) | 1.00 |  |
| Current User | 0.78 (0.37-1.59) | 0.500 |
| Previous User | 1.00 (0.39-2.61) | 0.995 |
| **24-month CBMP Use** |  |  |
| Oil (reference) | 1.00 |  |
| Dried flowers, Oils | 0.87 (0.35-2.02) | 0.743 |
| Dried flowers | 1.00 (0.39-2.47) | 1.000 |
| Other | 0.83 (0.24-3.00) | 0.774 |
| **24-month CBD Dose** |  |  |
| Minimum to Q1 (reference) | 1.00 |  |
| Q1 to Median | 1.18 (0.53-2.64) | 0.691 |
| Median to Q3 | 1.72 (0.74-4.13) | 0.217 |
| Q3 to Maximum | 1.08 (0.49-2.41) | 0.853 |
| **24-month THC Dose** |  |  |
| Minimum to Q1 (reference) | 1.00 |  |
| Q1 to Median | 0.94 (0.42-2.14) | 0.891 |
| Median to Q3 | 1.25 (0.53-2.96) | 0.607 |
| Q3 to Maximum | 0.92 (0.40-2.08) | 0.835 |
| **Baseline SQS Score** |  |  |
| Good-Excellent: 7-10 (reference) | 1.00 |  |
| Fair: 4-6 | 4.53 (2.15-9.90) | <0.001 |
| Terrible - Poor: 0-3 | 2.28 (1.10-4.81) | 0.028 |
| **Baseline GAD-7 Score** |  |  |
| Less than 5 (reference) | 1.00 |  |
| 5-9 | 1.34 (0.66-2.76) | 0.417 |
| 10-14 | 2.05 (0.86-5.19) | 0.115 |
| 15+ | 5.22 (1.98-16.52) | 0.002 |

**Supplementary Table 24: Univariable regression results for achieving a MCID in the MIDAS at 24-months.** MCID – minimal clinically important difference, MIDAS – migraine disability assessment test, CI – confidence interval, BMI – body mass index, CBMP – cannabis-based medicinal product, CBD – cannabidiol, THC – tetrahydrocannabinol, SQS – single item sleep quality scale, GAD-7- generalised anxiety disorder 7. GAD-7 Scoring: less than 5 – no anxiety symptoms, 5-9 – mild anxiety symptoms, 10-14 – moderate anxiety symptoms, 15+ - severe anxiety symptoms.

| **Variable** | **Odds Ratio (95% CI)** | **p-value** |
| --- | --- | --- |
| **Age** |  |  |
| Under 30 (reference) | 1.00 |  |
| 31-40 | 0.98 (0.43-2.19) | 0.959 |
| 41-50 | 0.93 (0.40-2.12) | 0.867 |
| 51-60 | 1.37 (0.39-5.67) | 0.634 |
| Over 60 | 1.50 (0.38-7.56) | 0.585 |
| **Sex** |  |  |
| Male (reference) | 1.00 |  |
| Female | 0.79 (0.44-1.42) | 0.430 |
| **BMI** |  |  |
| 20-24.99 (reference) | 1.00 |  |
| Under 20 | 0.90 (0.31-2.71) | 0.843 |
| 25-29.99 | 1.92 (0.86-4.47) | 0.119 |
| 30-34.99 | 0.90 (0.37-2.28) | 0.828 |
| 35+ | 0.65 (0.26-1.65) | 0.366 |
| **Cannabis Status** |  |  |
| Never Used (reference) | 1.00 |  |
| Current User | 0.78 (0.37-1.59) | 0.500 |
| Previous User | 1.00 (0.39-2.61) | 0.995 |
| **24-month CBMP Use** |  |  |
| Oil (reference) | 1.00 |  |
| Dried flowers, Oils | 0.87 (0.35-2.02) | 0.743 |
| Dried flowers | 1.00 (0.39-2.47) | 1.000 |
| Other | 0.83 (0.24-3.00) | 0.774 |
| **24-month CBD Dose** |  |  |
| Minimum to Q1 (reference) | 1.00 |  |
| Q1 to Median | 1.18 (0.53-2.64) | 0.691 |
| Median to Q3 | 1.72 (0.74-4.13) | 0.217 |
| Q3 to Maximum | 1.08 (0.49-2.41) | 0.853 |
| **24-month THC Dose** |  |  |
| Minimum to Q1 (reference) | 1.00 |  |
| Q1 to Median | 0.94 (0.42-2.14) | 0.891 |
| Median to Q3 | 1.25 (0.53-2.96) | 0.607 |
| Q3 to Maximum | 0.92 (0.40-2.08) | 0.835 |
| **Baseline SQS Score** |  |  |
| Good-Excellent: 7-10 (reference) | 1.00 |  |
| Fair: 4-6 | 4.53 (2.15-9.90) | <0.001 |
| Terrible - Poor: 0-3 | 2.28 (1.10-4.81) | 0.028 |
| **Baseline GAD-7 Score** |  |  |
| Less than 5 (reference) | 1.00 |  |
| 5-9 | 1.34 (0.66-2.76) | 0.417 |
| 10-14 | 2.05 (0.86-5.19) | 0.115 |
| 15+ | 5.22 (1.98-16.52) | 0.002 |

**Supplementary Table 25: Univariable regression results for achieving a positive improvement in the EQ-5D-5L at 24-months.** EQ-5D-5L – EuroQol 5-dimensions 5-levels, CI – confidence interval, BMI – body mass index, CBMP – cannabis-based medicinal product, CBD – cannabidiol, THC – tetrahydrocannabinol, SQS – single item sleep quality scale, GAD-7- generalised anxiety disorder 7. GAD-7 Scoring: less than 5 – no anxiety symptoms, 5-9 – mild anxiety symptoms, 10-14 – moderate anxiety symptoms, 15+ - severe anxiety symptoms.

| **Variable** | **Odds Ratio (95% CI)** | **p-value** |
| --- | --- | --- |
| **Age** |  |  |
| Under 30 (reference) | 1.00 |  |
| 31-40 | 1.06 (0.36-3.01) | 0.916 |
| 41-50 | 0.85 (0.30-2.31) | 0.745 |
| 51-60 | 1.40 (0.31-7.00) | 0.668 |
| Over 60 | 2.05 (0.38-13.52) | 0.424 |
| **Sex** |  |  |
| Male (reference) | 1.00 |  |
| Female | 0.80 (0.37-1.74) | 0.570 |
| **BMI** |  |  |
| 20-24.99 (reference) | 1.00 |  |
| Under 20 | 0.99 (0.27-3.70) | 0.987 |
| 25-29.99 | 2.91 (1.12-7.90) | 0.031 |
| 30-34.99 | 1.12 (0.38-3.32) | 0.840 |
| 35+ | 0.60 (0.19-1.83) | 0.366 |
| **Cannabis Status** |  |  |
| Never Used (reference) | 1.00 |  |
| Current User | 0.94 (0.34-2.52) | 0.903 |
| Previous User | 1.60 (0.49-5.43) | 0.440 |
| **24-month CBMP Use** |  |  |
| Oil (reference) | 1.00 |  |
| Dried flowers, Oils | 0.90 (0.20-4.08) | 0.894 |
| Dried flowers | 2.46 (0.44-14.77) | 0.311 |
| Other | 1.01 (0.16-6.63) | 0.991 |
| **24-month CBD Dose** |  |  |
| Minimum to Q1 (reference) | 1.00 |  |
| Q1 to Median | 3.48 (1.16-11.07) | 0.029 |
| Median to Q3 | 4.60 (1.40-16.08) | 0.014 |
| Q3 to Maximum | 1.82 (0.59-5.78) | 0.299 |
| **24-month THC Dose** |  |  |
| Minimum to Q1 (reference) | 1.00 |  |
| Q1 to Median | 0.71 (0.17-2.82) | 0.629 |
| Median to Q3 | 1.09 (0.27-4.21) | 0.906 |
| Q3 to Maximum | 0.72 (0.18-2.79) | 0.641 |
| **Baseline SQS Score** |  |  |
| Good-Excellent: 7-10 (reference) | 1.00 |  |
| Fair: 4-6 | 3.62 (1.44-9.46) | 0.007 |
| Terrible - Poor: 0-3 | 2.43 (1.02-5.96) | 0.048 |
| **Baseline GAD-7 Score** | |  |
| Less than 5 (reference) | 1.00 |  |
| 5-9 | 1.14 (0.48-2.72) | 0.771 |
| 10-14 | 1.59 (0.54-4.89) | 0.409 |
| 15+ | 4.65 (1.35-18.49) | 0.020 |

**Supplementary Table 26: Multivariable regression results for achieving a positive improvement in the EQ-5D-5L at 24-months.** EQ-5D-5L – EuroQol 5-dimensions 5-levels, CI – confidence interval, BMI – body mass index, CBMP – cannabis-based medicinal product, CBD – cannabidiol, THC – tetrahydrocannabinol, SQS – single item sleep quality scale, GAD-7- generalised anxiety disorder 7. GAD-7 Scoring: less than 5 – no anxiety symptoms, 5-9 – mild anxiety symptoms, 10-14 – moderate anxiety symptoms, 15+ - severe anxiety symptoms.

| **Variable** | **Odds Ratio (95% CI)** | **p-value** |
| --- | --- | --- |
| **Age** |  |  |
| Under 30 (reference) | 1 |  |
| 31-40 | 0.79 (0.36-1.71) | 0.546 |
| 41-50 | 0.61 (0.27-1.35) | 0.220 |
| 51-60 | 0.61 (0.16-2.02) | 0.425 |
| Over 60 | 1.21 (0.33-4.47) | 0.771 |
| **Sex** |  |  |
| Male (reference) | 1 |  |
| Female | 1.12 (0.63-1.98) | 0.692 |
| **BMI** |  |  |
| 20-24.99 (reference) |  |  |
| Under 20 | 0.38 (0.11-1.12) | 0.094 |
| 25-29.99 | 0.55 (0.26-1.14) | 0.109 |
| 30-34.99 | 0.39 (0.14-0.97) | 0.049 |
| 35+ | 0.99 (0.40-2.44) | 0.980 |
| **Cannabis Status** |  |  |
| Never Used (reference) |  |  |
| Current User | 0.69 (0.35-1.37) | 0.288 |
| Previous User | 0.55 (0.22-1.32) | 0.185 |
| **24-month CBMP Use** |  |  |
| Oil (reference) |  |  |
| Dried flowers, Oils | 1.78 (0.76-4.47) | 0.196 |
| Dried flowers | 1.86 (0.76-4.84) | 0.183 |
| Other | 1.39 (0.38-4.91) | 0.605 |
| **24-month CBD Dose** |  |  |
| Minimum to Q1 |  |  |
| Q1 to Median | 0.86 (0.39-1.89) | 0.714 |
| Median to Q3 | 0.99 (0.44-2.19) | 0.974 |
| Q3 to Maximum | 0.86 (0.39-1.89) | 0.714 |
| **24-month THC Dose** |  |  |
| Minimum to Q1 |  |  |
| Q1 to Median | 1.34 (0.61-3.00) | 0.466 |
| Median to Q3 | 1.37 (0.61-3.10) | 0.439 |
| Q3 to Maximum | 1.00 (0.44-2.26) | 1.000 |
| **Baseline SQS Score** |  |  |
| Good-Excellent: 7-10 (reference) | |  |
| Fair: 4-6 | 4.48 (2.11-10.05) | <0.001 |
| Terrible - Poor: 0-3 | 1.75 (0.78-4.06) | 0.179 |
| **Baseline GAD-7 Score** |  |  |
| Less than 5 (reference) |  |  |
| 5-9 | 11.12 (3.88-40.39) | <0.001 |
| 10-14 | 31.50 (10.06-123.55) | <0.001 |
| 15+ | 99.00 (29.16-432.59) | <0.001 |

**Supplementary Table 27: Univariable regression results for achieving a MCID in the GAD-7 at 24-months.** MCID – minimal clinically important difference, GAD-7- generalised anxiety disorder 7, CI – confidence interval, BMI – body mass index, CBMP – cannabis-based medicinal product, CBD – cannabidiol, THC – tetrahydrocannabinol, SQS – single item sleep quality scale. GAD-7 Scoring: less than 5 – no anxiety symptoms, 5-9 – mild anxiety symptoms, 10-14 – moderate anxiety symptoms, 15+ - severe anxiety symptoms.

| **Variable** | **Odds Ratio (95% CI)** | **p-value** |
| --- | --- | --- |
| **Age** |  |  |
| Under 30 (reference) | 1.00 |  |
| 31-40 | 0.69 (0.20-2.34) | 0.551 |
| 41-50 | 0.53 (0.16-1.74) | 0.298 |
| 51-60 | 1.45 (0.22-9.08) | 0.696 |
| Over 60 | 0.98 (0.15-6.74) | 0.981 |
| **Sex** |  |  |
| Male (reference) | 1.00 |  |
| Female | 0.50 (0.19-1.23) | 0.136 |
| **BMI** |  |  |
| 20-24.99 (reference) | 1.00 |  |
| Under 20 | 0.30 (0.05-1.45) | 0.146 |
| 25-29.99 | 0.48 (0.16-1.38) | 0.178 |
| 30-34.99 | 0.74 (0.20-2.74) | 0.654 |
| 35+ | 0.70 (0.19-2.58) | 0.594 |
| **Cannabis Status** |  |  |
| Never Used (reference) | 1.00 |  |
| Current User | 1.03 (0.32-3.26) | 0.955 |
| Previous User | 0.53 (0.13-2.06) | 0.367 |
| **24-month CBMP Use** |  |  |
| Oil (reference) |  |  |
| Dried flowers, Oils | 4.81 (0.78-32.63) | 0.096 |
| Dried flowers | 9.05 (1.06-84.91) | 0.047 |
| Other | 4.19 (0.49-40.41) | 0.197 |
| **24-month CBD Dose** |  |  |
| Minimum to Q1 | 1.00 |  |
| Q1 to Median | 2.00 (0.52-7.95) | 0.317 |
| Median to Q3 | 2.48 (0.61-10.57) | 0.209 |
| Q3 to Maximum | 1.34 (0.34-5.37) | 0.679 |
| **24-month THC Dose** |  |  |
| Minimum to Q1 | 1.00 |  |
| Q1 to Median | 0.47 (0.08-2.51) | 0.377 |
| Median to Q3 | 0.48 (0.08-2.58) | 0.402 |
| Q3 to Maximum | 0.64 (0.11-3.56) | 0.609 |
| **Baseline SQS Score** |  |  |
| Good-Excellent: 7-10 (reference) | 1.00 |  |
| Fair: 4-6 | 1.27 (0.39-4.12) | 0.686 |
| Terrible - Poor: 0-3 | 1.04 (0.33-3.30) | 0.950 |
| **Baseline GAD-7 Score** |  |  |
| Less than 5 (reference) | 1.00 |  |
| 5-9 | 14.58 (4.32-62.51) | <0.001 |
| 10-14 | 51.75 (12.71-271.79) | <0.001 |
| 15+ | 167.26 (34.67-1098.00) | <0.001 |

**Supplementary Table 28: Multivariable regression results for achieving a MCID in the GAD-7 at 24-months.** MCID – minimal clinically important difference, GAD-7- generalised anxiety disorder 7, CI – confidence interval, BMI – body mass index, CBMP – cannabis-based medicinal product, CBD – cannabidiol, THC – tetrahydrocannabinol, SQS – single item sleep quality scale. GAD-7 Scoring: less than 5 – no anxiety symptoms, 5-9 – mild anxiety symptoms, 10-14 – moderate anxiety symptoms, 15+ - severe anxiety symptoms.

| **Variable** | **Odds Ratio (95% CI)** | **p-value** |
| --- | --- | --- |
| **Age** |  |  |
| Under 30 (reference) | 1.00 |  |
| 31-40 | 1.01 (0.47-2.18) | 0.978 |
| 41-50 | 1.07 (0.49-2.34) | 0.873 |
| 51-60 | 0.55 (0.15-1.83) | 0.342 |
| Over 60 | 1.10 (0.30-4.06) | 0.884 |
| **Sex** |  |  |
| Male (reference) | 1.00 |  |
| Female | 1.17 (0.67-2.03) | 0.588 |
| **BMI** |  |  |
| 20-24.99 (reference) | 1.00 |  |
| Under 20 | 2.29 (0.82-6.70) | 0.119 |
| 25-29.99 | 1.79 (0.87-3.74) | 0.117 |
| 30-34.99 | 1.11 (0.45-2.69) | 0.816 |
| 35+ | 2.08 (0.84-5.27) | 0.115 |
| **Cannabis Status** |  |  |
| Never Used (reference) | 1.00 |  |
| Current User | 0.32 (0.15-0.64) | 0.002 |
| Previous User | 0.20 (0.08-0.50) | <0.001 |
| **24-month CBMP Use** |  |  |
| Oil (reference) | 1.00 |  |
| Dried flowers, Oils | 1.14 (0.51-2.58) | 0.746 |
| Dried flowers | 0.56 (0.24-1.33) | 0.188 |
| Other | 1.13 (0.34-3.72) | 0.845 |
| **24-month CBD Dose** |  |  |
| Minimum to Q1 | 1.00 |  |
| Q1 to Median | 1.19 (0.55-2.60) | 0.652 |
| Median to Q3 | 1.28 (0.58-2.83) | 0.540 |
| Q3 to Maximum | 1.92 (0.89-4.21) | 0.100 |
| **24-month THC Dose** |  |  |
| Minimum to Q1 | 1.00 |  |
| Q1 to Median | 1.21 (0.56-2.64) | 0.624 |
| Median to Q3 | 0.85 (0.38-1.86) | 0.680 |
| Q3 to Maximum | 0.73 (0.33-1.59) | 0.427 |
| **Baseline SQS Score** |  |  |
| Good-Excellent: 7-10 (reference) | |  |
| Fair: 4-6 | 28.57 (10.88-91.01) | <0.001 |
| Terrible - Poor: 0-3 | 8.61 (3.29-27.16) | <0.001 |
| **Baseline GAD-7 Score** |  |  |
| Less than 5 (reference) | 1.00 |  |
| 5-9 | 1.75 (0.86-3.57) | 0.121 |
| 10-14 | 1.93 (0.84-4.46) | 0.120 |
| 15+ | 3.24 (1.47-7.40) | 0.004 |

**Supplementary Table 29: Univariable regression results for achieving a MCID in the SQS at 24-months.** MCID – minimal clinically important difference, SQS – single item sleep quality scale, CI – confidence interval, BMI – body mass index, CBMP – cannabis-based medicinal product, CBD – cannabidiol, THC – tetrahydrocannabinol, GAD-7- generalised anxiety disorder 7. GAD-7 Scoring: less than 5 – no anxiety symptoms, 5-9 – mild anxiety symptoms, 10-14 – moderate anxiety symptoms, 15+ - severe anxiety symptoms.

| **Variable** | **Odds Ratio (95% CI)** | **p-value** |
| --- | --- | --- |
| **Age** |  |  |
| Under 30 (reference) | 1.00 |  |
| 31-40 | 0.49 (0.14-1.59) | 0.242 |
| 41-50 | 0.89 (0.27-2.84) | 0.842 |
| 51-60 | 0.10 (0.01-0.56) | 0.011 |
| Over 60 | 2.14 (0.28-15.81) | 0.452 |
| **Sex** |  |  |
| Male (reference) | 1.00 |  |
| Female | 1.36 (0.56-3.37) | 0.497 |
| **BMI** |  |  |
| 20-24.99 (reference) | 1.00 |  |
| Under 20 | 4.15 (0.95-19.59) | 0.063 |
| 25-29.99 | 3.52 (1.19-10.97) | 0.025 |
| 30-34.99 | 1.00 (0.28-3.53) | 1.000 |
| 35+ | 4.19 (1.09-17.76) | 0.042 |
| **Cannabis Status** |  |  |
| Never Used (reference) | 1.00 |  |
| Current User | 0.36 (0.11-1.12) | 0.086 |
| Previous User | 0.17 (0.04-0.60) | 0.008 |
| **24-month CBMP Use** |  |  |
| Oil (reference) | 1.00 |  |
| Dried flowers, Oils | 1.03 (0.16-6.20) | 0.976 |
| Dried flowers | 0.36 (0.04-2.68) | 0.322 |
| Other | 3.58 (0.38-34.60) | 0.261 |
| **24-month CBD Dose** |  |  |
| Minimum to Q1 (reference) | 1.00 |  |
| Q1 to Median | 1.37 (0.38-5.05) | 0.631 |
| Median to Q3 | 0.37 (0.09-1.39) | 0.148 |
| Q3 to Maximum | 1.16 (0.33-4.12) | 0.813 |
| **24-month THC Dose** |  |  |
| Minimum to Q1 (reference) | 1.00 |  |
| Q1 to Median | 0.99 (0.18-5.41) | 0.986 |
| Median to Q3 | 0.45 (0.08-2.45) | 0.357 |
| Q3 to Maximum | 0.60 (0.11-3.36) | 0.563 |
| **Baseline SQS Score** |  |  |
| Good-Excellent: 7-10 (reference) | 1.00 |  |
| Fair: 4-6 | 162.97 (35.88-1025.85) | <0.001 |
| Terrible - Poor: 0-3 | 23.37 (6.25-112.39) | <0.001 |
| **Baseline GAD-7 Score** |  |  |
| Less than 5 (reference) | 1.00 |  |
| 5-9 | 0.83 (0.28-2.46) | 0.743 |
| 10-14 | 0.25 (0.07-0.88) | 0.035 |
| 15+ | 0.84 (0.24-2.93) | 0.783 |

**Supplementary Table 30: Multivariable regression results for achieving a MCID in the SQS at 24-months.** MCID – minimal clinically important difference, SQS – single item sleep quality scale, CI – confidence interval, BMI – body mass index, CBMP – cannabis-based medicinal product, CBD – cannabidiol, THC – tetrahydrocannabinol, GAD-7- generalised anxiety disorder 7. GAD-7 Scoring: less than 5 – no anxiety symptoms, 5-9 – mild anxiety symptoms, 10-14 – moderate anxiety symptoms, 15+ - severe anxiety symptoms.

| **Variable** | **Odds Ratio (95% CI)** | **p-value** |
| --- | --- | --- |
| **Age** |  |  |
| Under 30 (reference) | 1.00 |  |
| 31-40 | 1.56 (0.48-6.00) | 0.480 |
| 41-50 | 2.71 (0.89-10.18) | 0.100 |
| 51-60 | not estimable* | - |
| Over 60 | 3.17 (0.55-17.03) | 0.175 |
| **Sex** |  |  |
| Male (reference) | 1.00 |  |
| Female | 1.97 (0.91-4.36) | 0.087 |
| **BMI** |  |  |
| 20-24.99 (reference) | 1.00 |  |
| Under 20 | 3.45 (0.88-13.15) | 0.066 |
| 25-29.99 | 1.16 (0.34-3.93) | 0.807 |
| 30-34.99 | 1.93 (0.51-7.01) | 0.311 |
| 35+ | 2.76 (0.79-9.77) | 0.107 |
| **Cannabis Status** |  |  |
| Never Used (reference) | 1.00 |  |
| Current User | 0.57 (0.24-1.42) | 0.215 |
| Previous User | 0.58 (0.17-1.81) | 0.360 |
| **24-month CBMP Use** |  |  |
| Oil (reference) | 1.00 |  |
| Dried flowers, Oils | 3.49 (0.92-22.89) | 0.108 |
| Dried flowers | 2.45 (0.59-16.79) | 0.270 |
| Other | 3.21 (0.48-26.56) | 0.228 |
| **24-month CBD Dose** |  |  |
| Minimum to Q1 (reference) | 1.00 |  |
| Q1 to Median | 0.37 (0.10-1.21) | 0.118 |
| Median to Q3 | 0.52 (0.15-1.60) | 0.272 |
| Q3 to Maximum | 1.35 (0.53-3.54) | 0.529 |
| **24-month THC Dose** |  |  |
| Minimum to Q1 (reference) | 1.00 |  |
| Q1 to Median | 0.57 (0.19-1.58) | 0.282 |
| Median to Q3 | 0.51 (0.16-1.46) | 0.220 |
| Q3 to Maximum | 0.58 (0.20-1.61) | 0.302 |
| **Baseline SQS Score** |  |  |
| Good-Excellent: 7-10 (reference) | 1.00 |  |
| Fair: 4-6 | 1.19 (0.45-3.41) | 0.731 |
| Terrible - Poor: 0-3 | 1.50 (0.56-4.30) | 0.434 |
| **Baseline GAD-7 Score** | |  |
| Less than 5 (reference) | 1.00 |  |
| 5-9 | 0.86 (0.30-2.35) | 0.775 |
| 10-14 | 2.22 (0.80-6.03) | 0.118 |
| 15+ | 0.68 (0.18-2.14) | 0.527 |

**Supplementary Table 31: Univariable regression results for the occurrence of adverse events.** MCID – minimal clinically important difference, SQS – single item sleep quality scale, CI – confidence interval, BMI – body mass index, CBMP – cannabis-based medicinal product, CBD – cannabidiol, THC – tetrahydrocannabinol, GAD-7- generalised anxiety disorder 7. GAD-7 Scoring: less than 5 – no anxiety symptoms, 5-9 – mild anxiety symptoms, 10-14 – moderate anxiety symptoms, 15+ - severe anxiety symptoms. * - Odds ratio could not be estimated due to extreme sparsity of data.

| **Variable** | **Odds Ratio (95% CI)** | **p-value** |
| --- | --- | --- |
| **Age** |  |  |
| Under 30 (reference) | 1.00 |  |
| 31-40 | 0.50 (0.11-2.48) | 0.380 |
| 41-50 | 1.05 (0.24-5.05) | 0.949 |
| 51-60 | not estimable* | - |
| Over 60 | 1.35 (0.15-11.24) | 0.781 |
| **Sex** |  |  |
| Male (reference) | 1.00 |  |
| Female | 1.51 (0.51-4.61) | 0.460 |
| **BMI** |  |  |
| 20-24.99 (reference) | 1.00 |  |
| Under 20 | 4.67 (0.86-26.17) | 0.073 |
| 25-29.99 | 1.38 (0.30-6.55) | 0.681 |
| 30-34.99 | 1.64 (0.32-8.23) | 0.544 |
| 35+ | 9.21 (1.80-54.34) | 0.010 |
| **Cannabis Status** |  |  |
| Never Used (reference) | 1.00 |  |
| Current User | 0.45 (0.12-1.68) | 0.228 |
| Previous User | 0.33 (0.05-1.77) | 0.214 |
| **24-month CBMP Use** |  |  |
| Oil (reference) | 1.00 |  |
| Dried flowers, Oils | 21.29 (2.70-261.35) | 0.007 |
| Dried flowers | 18.24 (1.74-276.04) | 0.022 |
| Other | 10.75 (0.58-231.87) | 0.110 |
| **24-month CBD Dose** |  |  |
| Minimum to Q1 (reference) | 1.00 |  |
| Q1 to Median | 0.19 (0.03-1.01) | 0.061 |
| Median to Q3 | 0.19 (0.03-1.13) | 0.075 |
| Q3 to Maximum | 0.56 (0.12-2.43) | 0.443 |
| **24-month THC Dose** |  |  |
| Minimum to Q1 (reference) | 1.00 |  |
| Q1 to Median | 0.08 (0.01-0.44) | 0.006 |
| Median to Q3 | 0.12 (0.02-0.62) | 0.013 |
| Q3 to Maximum | 0.17 (0.03-0.87) | 0.038 |
| **Baseline SQS Score** |  |  |
| Good-Excellent: 7-10 (reference) | 1.00 |  |
| Fair: 4-6 | 1.33 (0.33-5.45) | 0.685 |
| Terrible - Poor: 0-3 | 2.22 (0.61-8.91) | 0.237 |
| **Baseline GAD-7 Score** |  |  |
| Less than 5 (reference) | 1.00 |  |
| 5-9 | 0.79 (0.21-2.82) | 0.722 |
| 10-14 | 2.21 (0.55-9.00) | 0.259 |
| 15+ | 0.16 (0.02-0.94) | 0.058 |

**Supplementary Table 32: Univariable regression results for the occurrence of adverse events.** MCID – minimal clinically important difference, SQS – single item sleep quality scale, CI – confidence interval, BMI – body mass index, CBMP – cannabis-based medicinal product, CBD – cannabidiol, THC – tetrahydrocannabinol, GAD-7- generalised anxiety disorder 7. GAD-7 Scoring: less than 5 – no anxiety symptoms, 5-9 – mild anxiety symptoms, 10-14 – moderate anxiety symptoms, 15+ - severe anxiety symptoms. * - Odds ratio could not be estimated due to extreme sparsity of data.
